# Acute Adenosine Receptor Antagonism in Combination With Acute Intermittent Hypoxia to Promote Breathing Plasticity in Amyotrophic Lateral Sclerosis: Protocol for a Randomized, Double-Blinded, Placebo-Controlled Trial

**DOI:** 10.2196/76105

**Published:** 2025-11-07

**Authors:** Priscila Sales de Campos, May Smith-Hublou, Wendy L Olsen, Wagner Souza Leite, James P Wymer, Nicholas J Napoli, Alicia K Vose, Michael T Pulley, Gordon S Mitchell, Barbara K Smith

**Affiliations:** 1 Breathing Research and Therapeutics Center University of Florida Gainesville, FL United States; 2 College of Public Health and Health Professions, Department of Physical Therapy University of Florida Gainesville, FL United States; 3 Department of Physical Therapy Center for Health and Sports Sciences Universidade do Estado de Santa Catarina Florianopolis Brazil; 4 McKnight Brain Institute University of Florida Gainesville, FL United States; 5 College of Engineering, Department of Electrical and Computer Engineering University of Florida Gainesville, FL United States; 6 Beaver College of Health Sciences, Department of Rehabilitation Sciences Appalachian State University Boone, NC United States; 7 College of Medicine, Department of Neurology University of Florida Gainesville, FL United States; 8 College of Medicine-Jacksonville, Department of Neurology University of Florida Jacksonville, FL United States; 9 College of Medicine, Department of Pediatrics University of Florida Gainesville, FL United States

**Keywords:** acute intermittent hypoxia, adenosine A2A receptor, amyotrophic lateral sclerosis, neuronal plasticity, respiration

## Abstract

**Background:**

Respiratory impairment is a major concern in amyotrophic lateral sclerosis (ALS), shortening survival and lowering quality of life. One therapy with promise to delay respiratory decline in ALS is acute intermittent hypoxia (AIH), consisting of alternating periods of breathing mildly hypoxic (9%-12% O_2_) and normoxic (21% O_2_) gas. AIH stimulates spinal, serotonin-dependent neuroplasticity in rodent models, conferring functional benefits in diverse physiological systems without detectable pathology. However, in rodent models, AIH-induced neuroplasticity is constrained by distinct signaling cascades initiated by spinal adenosine.

**Objective:**

We propose to investigate a therapeutic strategy to delay breathing compromise in those living with ALS by combining a selective adenosine 2A (A_2_A) receptor inhibitor (istradefylline) with AIH. The fundamental hypothesis guiding this proposal is that a single AIH trial after pretreatment with istradefylline enhances respiratory neuroplasticity versus AIH or sham intervention.

**Methods:**

We propose to evaluate resting breathing, respiratory strength, and participant-reported symptoms in adults living with ALS after combined istradefylline plus AIH. A mixed within- and between-participant study design incorporates 4 test sessions, separated by approximately 2 weeks (±5 days). Testing conditions include single sessions of AIH + istradefylline, AIH + placebo, sham AIH (ie, normoxia) + placebo, and sham AIH + istradefylline. Safety and feasibility will be characterized using the rate of adverse events, changes in vital signs, and participant-reported breathing sensations (Aim 1). Neuroplasticity of breathing and motor function will be evaluated as changes in resting breathing, voluntary respiratory strength, respiratory control, and maximal pinch force (Aim 2).

**Results:**

As of January 2025, with a target sample of 16 participants in each group, 10 participants with ALS and 5 control participants completed study procedures. Recruiting is ongoing, and the final participant will complete the study by December 2025. Publication of results is expected by the end of 2026.

**Conclusions:**

These aims will provide crucial data regarding the preliminary safety and feasibility of this paired intervention and help optimize therapeutic AIH as a rehabilitation strategy, thereby guiding further research concerning this novel treatment for ALS.

**Trial Registration:**

ClinicalTrials.gov NCT05377424; https://clinicaltrials.gov/study/NCT05377424

**International Registered Report Identifier (IRRID):**

DERR1-10.2196/76105

## Introduction

### Overview

Amyotrophic lateral sclerosis (ALS) is a progressive neurodegenerative disorder characterized by motor neuron death in the primary motor cortex, brain stem, and spinal cord, leading to muscular weakness and atrophy [1,2]. ALS can lead to progressive, variable weakness of the skeletal muscles involved in swallowing, speaking, coughing, limb movement, trunk control, and breathing. Respiratory muscle denervation causes diaphragm and accessory muscle weakness, hypoventilation, dyspnea, and orthopnea [3,4]. Ineffective airway clearance and aspiration result from bulbar denervation. Respiratory and bulbar dysfunction lead to the need for mechanical ventilation [5], shortened survival, and diminished quality of life [6]. Typical survival is 2-5 years post diagnosis [7]. Treatments that preserve or improve breathing would have a considerable impact on the duration and quality of life.

Acute intermittent hypoxia (AIH) consists of short, repeated exposures to mild or moderate hypoxia (9%-12% O_2_), separated by normoxic episodes (21% O_2_). Hypoxic episodes activate the carotid body chemoreceptors, sending chemoafferent signals to stimulate medullary respiratory neurons [8] as well as brain stem raphe neurons that contain the neurochemical serotonin. When this neural network is repeatedly stimulated during AIH [9], serotonin release from the raphe neurons triggers cell signaling pathways resulting in new synthesis of brain-derived neurotrophic factor protein. In turn, brain-derived neurotrophic factor strengthens synapses onto respiratory (and nonrespiratory) motor neurons [10-12], increasing respiratory motor output. AIH-induced motor plasticity was first described as a persistent enhancement of phrenic neural activity lasting hours after AIH has ended, an effect known as phrenic long-term facilitation (pLTF); a similar effect occurs in hypoglossal motor neurons [13,14]. pLTF is a form of activity-independent, neuromodulator-induced neuroplasticity that exhibits sensitivity to the pattern of hypoxic episodes and time of day [8,15,16]. Repeated AIH appears to amplify and prolong pLTF without detectable pathology [17-20], Thus, AIH may induce respiratory rehabilitation in ALS as well as other neurological conditions that compromise breathing [21].

Our group recently completed the first clinical trial of AIH administration in patients with ALS compared with unaffected controls. In this study, AIH enhanced tidal volume, minute ventilation, and collective respiratory muscle activation of participants living with ALS versus age- and sex-matched controls. Importantly, AIH was well-tolerated and free of treatment-related adverse effects; the participants typically could not distinguish between AIH versus sham AIH (ie, normoxia) [22].

**Figure 1:**
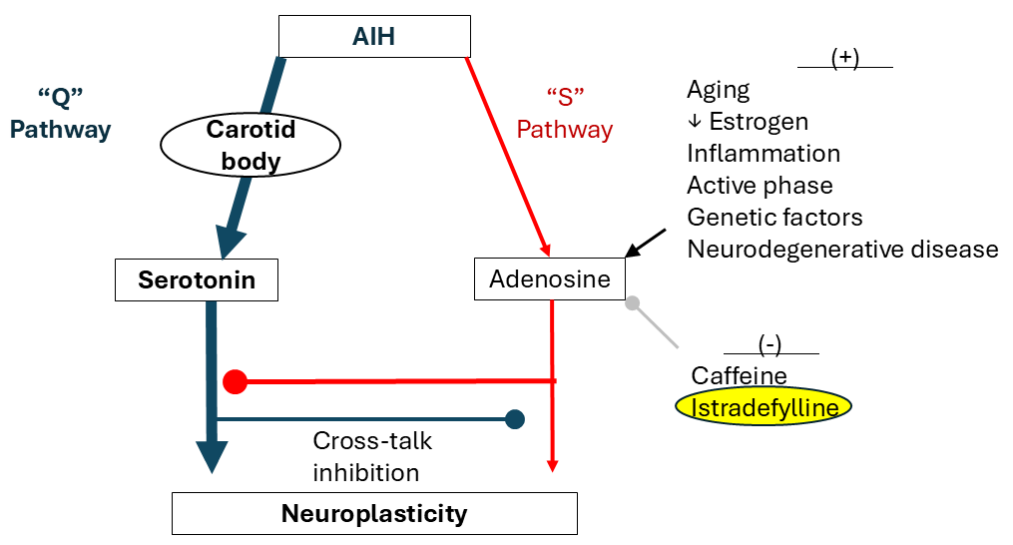
Competing serotonin- (Q) and adenosine-driven (S) pathways to phrenic motor facilitation. Listed on the right are factors that impact serotonin-dominant plasticity due to increased adenosinergic “S” pathway inhibition. By combining istradefylline with mild acute intermittent hypoxia (AIH), this protocol aims to minimize crosstalk inhibition by reducing S to Q pathway inhibition, allowing full expression of the “Q” pathway.

While the modest AIH used in rehabilitation typically elicits motor facilitation via a serotonin-dependent mechanism (Gq protein–coupled receptor signaling [termed the “Q” pathway]) [8,14,23,24], an alternative AIH-induced signaling pathway results from adenosine (Gs protein–coupled receptor signaling [termed the “S” pathway]) [25-28], acting on adenosine 2A (A_2_A) receptors [29]. Serotonin and adenosine signaling oppose one another through powerful cross-talk inhibition; in extreme cases, this “battle” cancels phrenic motor plasticity [28,30-32]. Although both serotonin and adenosine receptors can be activated during mild hypoxia, the serotonergic “Q” pathway predominates. However, multiple personal characteristics, disease states, and treatment factors upregulate the adenosinergic “S” pathway, sufficiently to inhibit plasticity (Figure 1), including aging [33], low estrogen [33-35], inflammation [36], active phase delivery [37], genetic factors [33], and traumatic or neurodegenerative diseases [38].

In rodent models of spinal cord injury, an increased pLTF was observed after the administration of paired AIH and the A_2_A receptor inhibitor istradefylline [31]. In a subsequent clinical study, the impact of combined AIH and caffeine, a nonselective adenosine antagonist, found the combined approach further improved walking speed and distance in people with chronic spinal injuries [39]. Thus, combined AIH and A_2_A receptor antagonism may amplify respiratory motor plasticity [40]. However, since caffeine is a nonselective adenosine antagonist that also inhibits phosphodiesterase action, off-target effects such as nausea and elevated heart rate (HR) could limit its use in the doses needed to augment AIH plasticity. This study uses istradefylline for its greater affinity for the A_2_A receptor and its tolerance in clinical studies of older adults and those with Parkinson disease (PD).

Elevated spinal adenosine is a characteristic of neurodegenerative diseases, such as ALS [41,42]. In the SOD1^G93A^ (copper zinc superoxide dismutase 1) mouse model of ALS, A_2_A receptor signaling contributed to motor neuron degeneration, while A_2_A receptor antagonism delayed disease progression [42]. A_2_A antagonism can also have neuroprotective and anti-inflammatory effects in motor neurons [43]. NOURIANZ (istradefylline) is a highly selective A_2_A receptor antagonist currently approved for use in adults with PD to offset inhibitory effects of upregulated A_2_A receptors on D2 dopamine receptors of the basal ganglia [44]. Istradefylline reduces daily “off” time in people with PD, improving periods of mobility, and is reported safe and well tolerated [45]. Dyskinesia (5%) and hallucinations (3.4%) were the most common side effects associated with longer-term use [46], but cognition, sedation, or risk of cardiovascular events were not impacted [47]. Considering the demonstrated safety of chronic istradefylline in PD and healthy controls, we hypothesize that this brief, temporary treatment will be safe in ALS.

We further hypothesize that the combination of istradefylline and AIH, in patients with ALS, will elicit greater gains in respiratory and nonrespiratory motor function than either intervention alone, and that will contribute to improvements in breathing and motor function. In a rodent model of phrenic motor neuron death, significant A_2_A receptor upregulation preceded motor neuron loss, but the coadministration of istradefylline improved motor neuron survival and maintained diaphragm function [43]. The information gained in this investigation will help us optimize intervention approaches for future studies of repeated, daily AIH as a rehabilitation tool. Thus, this study represents an important step in the iterative process of moving AIH protocols toward clinical practice.

### Aims

The objective of this study is to evaluate single and combined effects of selective A_2_A receptor antagonism and AIH on respiratory strength, resting breathing, and participant-reported symptoms in adults living with ALS and age-matched unaffected adults. The following two aims are proposed to test the hypotheses:

Aim 1: a single 20 mg istradefylline dose prior to AIH is safe and feasible. Dependent variables will include rate of adverse events (primary outcome), acute changes in vital signs (HR, respiratory rate, blood pressure [BP], etc), patient-reported symptoms (dyskinesias and dizziness), and study completion.

Aim 2: a single 20 mg istradefylline dose prior to AIH improves respiratory motor function when compared to either intervention alone. Dependent variables will include resting tidal volume (primary outcome), breathing rate and minute ventilation, respiratory muscle assessments via multichannel surface electromyography (sEMG), cough force, maximal inspiratory pressure (MIP), and maximal finger pinch force.

## Methods

### Study Design, Setting, and Recruitment

This is a repeated-measure, double-blinded, placebo-controlled, randomized trial, administered at a single site, the University of Florida (UF). This clinical trial was registered in the United States National Library of Medicine through ClinicalTrials.gov (NCT05377424).

Patients living with ALS will be recruited from the UF ALS clinic, as well as through flyers, word of mouth, and the study registry website. Patients who meet the study criteria will be identified by the study physicians through the UF adult neuromuscular clinic. Healthy age-matched controls will be recruited via advertising for the study on ClinicalTrials.gov, StudyConnect, social media for the UF Clinical and Translational Science Institute, and flyers throughout the local community. Additional potential healthy controls will be identified from the Pepper Center Registry maintained by the UF Institute on Aging, and institutional review board (IRB)–approved flyers will be sent to their address. Study interventions will occur at a university-based clinical research center located in Gainesville, FL.

### Inclusion Criteria

Eligible participants include nonsmoking adults diagnosed with ALS or age-matched unaffected controls, aged 21-80 years. Eligible participants will have El Escorial diagnostic classification of probable or definite ALS and vital capacity (VC) ≥60% of predicted value; we seek to treat participants before they approach respiratory failure and need for mechanical ventilation, which typically occurs when VC falls below 50% predicted [48]. The ALS Functional Rating Scale-Revised (ALSFRS-R) consists of 12 ordinal items of 5 points (rated 0-4) each, which describe impairments in different systems functionally, where a score of “4” reflects normal function and “0” reflects profound dysfunction [49]. Scores below 2 for respiratory and bulbar items typically reflect severe functional impairment (ie, need for supplemental tube feeding and continuous use of respiratory support). Clinically prescribed ALS medications must be at a stable dose for 30 days.

### Exclusion Criteria

Participants (both affected and controls) are ineligible if they are pregnant, have an active respiratory infection, took antibiotics within 4 weeks of recruitment, are diagnosed with another neurodegenerative disease, have symptomatic cardiovascular disease or dysrhythmias, have a BMI of >35 kg/m^2^, have a seizure disorder, use respiratory inhalers daily for airway disease, or require external respiratory support while awake and upright. In addition, the following conditions are exclusionary for the use of istradefylline: routine use of CYP3A4 inducers (eg, carbamazepine, rifampin, phenytoin, and St John’s Wort), history of moderate renal impairment or severe hepatic impairment, and history of hallucinations or psychosis.

### Rationale for Including Age-Matched Controls

AIH has shown to improve cognitive and exercise performance by improving hemodynamics and work capacity in healthy older adults [50,51]. While effects of AIH and istradefylline have been tested separately in healthy older adults, there is a lack of evidence regarding the additive effects of pairing istradefylline with AIH. Since older adults are the individuals who typically have the greatest need for rehabilitation, we are interested to test whether combined istradefylline and AIH can augment respiratory motor plasticity in healthy older adults.

Healthy older adults have been previously included in clinical trials to evaluate the exposure-response relationship and pharmacokinetic disposition of istradefylline in patients with PD [45,52]. By including healthy older adults in our study, we can determine whether any observed differences with paired AIH and istradefylline among individuals with ALS are specific to ALS or are potentially more generalizable to the older adult population.

### Study Procedures

The SPIRIT (Standard Protocol Items: Recommendations for Interventional Trials) flow chart with all study procedures can be found in Table 1.

**Table 1:** SPIRIT (Standard Protocol Items: Recommendations for Interventional Trials) flow chart with a schedule of enrollment, interventions, and assessments.

Activity			Study period												
			Enrollment		Allocation		Postallocation visits separated by 2 weeks (±5 days), 6-10 weeks total								2-week follow-up
			T–1		T0		T1	T2		T3		T4		T5	
**Enrollment**															
	Eligibility screen	✓													
	Informed consent	✓													
	Allocation			✓											
**Interventions**															
	Istradefylline + AIH^a^					—^b^		—	—		—				
	Istradefylline + sham AIH					—		—	—		—				
	Placebo + AIH					—		—	—		—				
	Placebo + sham AIH					—		—	—		—				
**Assessments**															
	Study completion rate												✓		
	Adverse events					✓		✓	✓		✓		✓		
	Vital signs (HR^c^/BP^d^)					✓		✓	✓		✓				
	Participant-reported symptoms					✓		✓	✓		✓				
	Borg dyspnea scale					✓		✓	✓		✓				
	Laboratory tests					✓		✓	✓		✓				
	Minute ventilation					✓		✓	✓		✓				
	MIF^e^					✓		✓	✓		✓				
	PCF^f^					✓		✓	✓		✓				
	MIP^g^					✓		✓	✓		✓				
	SNIP^h^					✓		✓	✓		✓				
	Pinch force					✓		✓	✓		✓				

^a^AIH: acute intermittent hypoxia.

^b^Administered at 1 visit, in randomized order.

^c^HR: heart rate.

^d^BP: blood pressure.

^e^MIP: maximal inspiratory pressure.

^f^PCF: peak cough flow.

^g^MIP: maximal inspiratory pressure.

^h^SNIP: sniff nasal inspiratory pressure.

### Ethical Considerations

Ethical approval was granted by the UF IRB (IRB 202101568) on May 16, 2022. The US Food and Drug Administration provided clearance for off-label investigational use of istradefylline as described in the protocol (investigational new drug application, approved April 8, 2022; 156267). Informed consent will be obtained in writing from all participants prior to enrollment by study team members. See Multimedia Appendices 1 and 2 for informed consent forms. Participants were informed they would be able to withdraw from the study at any time for any reason. This work is supported by the ALS Association. Outside of the human research protection review, the funding sponsor does not have a direct role in directing the study design; collection, management, analysis, and interpretation of data; writing of the report; or the decision to submit the report for publication.

Health information will be collected, used, and shared with others (ie, clinical and research staff and the UF Research Participant Payment team) to determine if interested individuals are eligible to participate, and then as part of the participation in the study. The following information may be collected, used, and shared with others: medical history, including past conditions and surgeries, medications, and information related to diagnosis of ALS; social security number (for reimbursement purposes); demographic information; contact information; and the results of the research tests done for this study. The research team may collect this information from other health care providers, such as laboratories, which are a part of this research, as well as health care providers that are not part of this research (other doctors, hospitals, or clinics). Other professionals at the UF or UF Health Hospital who provide study-related care, and the UF IRB, may also collect health information. Participants will be informed of the collection and protection of health information collected as part of the informed consent process. Health information and study data will be stored in locked filing cabinets or on computer servers with secure passwords or encrypted electronic storage devices, as required by UF policy.

Participants will be compensated US $20 for the screening visit and US $120 for each treatment visit, via prepaid Visa gift cards. Additionally, overnight hotel expenses will be covered by the study for participants traveling from out of town, and lunch will be provided during treatment visits.

### Screening

**Figure 2:**
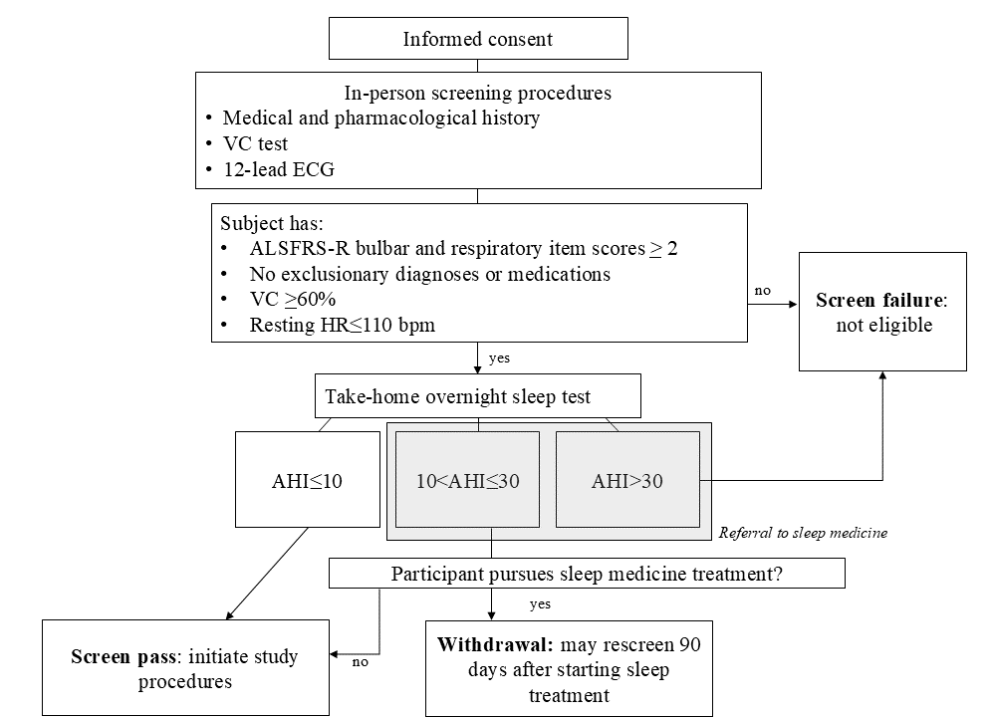
Screening procedures. AHI: apnea-hypopnea index; ALSFRS-R: Amyotrophic Lateral Sclerosis Functional Rating Scale-Revised; ECG: electrocardiogram; HR: heart rate; VC: vital capacity.

Interested individuals who meet the basic study criteria, agree to participate, and sign the IRB-approved informed consent form will undergo further screening procedures prior to the first interventional visit. Prospective participants will be informed of the eligibility criteria, study procedures, participation risks, privacy and safety precautions in place, alternatives to study participation, and the right to withdraw from the study at any time. The study coordinator will schedule visits and communicate with participants by phone and email. The study coordinator or a designated investigator will complete consenting and screening tests (Figure 2).

Screening tests shall include:

1. Patient demographic information and medical history: sociodemographic (age and sex), anthropometric (height and weight to calculate the BMI), and general clinical data (medical history, comorbidities, and medications).
2. VC: the maximal volume of gas that can be exhaled, following full inspiration. Upright VC will be administered per American Thoracic Society and European Respiratory Society guidelines [53,54]. Individuals with less than 60% of predicted VC will be ineligible for further participation in this study.
3. 12-lead electrocardiogram: cardiac rate and rhythm will be evaluated. The presence of resting tachycardia (HR >110 bpm), atrial fibrillation, or other dysrhythmias will exclude people from participation.
4. STOP-BANG (Snoring, Tiredness, Observed Apnea, High BP-BMI, Age, Neck Circumference, and Gender) questionnaire: questionnaire to estimate the risk of obstructive sleep apnea (OSA) [55]. A meta-analysis found a STOP-BANG score of ≥3 had excellent sensitivity and high discriminatory power to exclude moderate to severe OSA [56].
5. Overnight sleep monitor: participants will wear an overnight sensor that noninvasively monitors arterial oximetry and breathing parameters (Nox T3s; Nox Medical) to estimate the severity of sleep-disordered breathing [57]. If clinically significant OSA (apnea-hypopnea index [AHI] >10 events/hour) is detected, participants will be offered a referral to a clinical sleep specialist for further evaluation. If they choose to pursue clinical treatment for sleep apnea, they will be withdrawn and eligible to rescreen for participation after receiving 3 months of treatment. If they choose not to seek treatment, they will be included in the study. Participants with severe OSA AHI >30 events/hour will be ineligible.

### Study Visits

**Figure 3:**
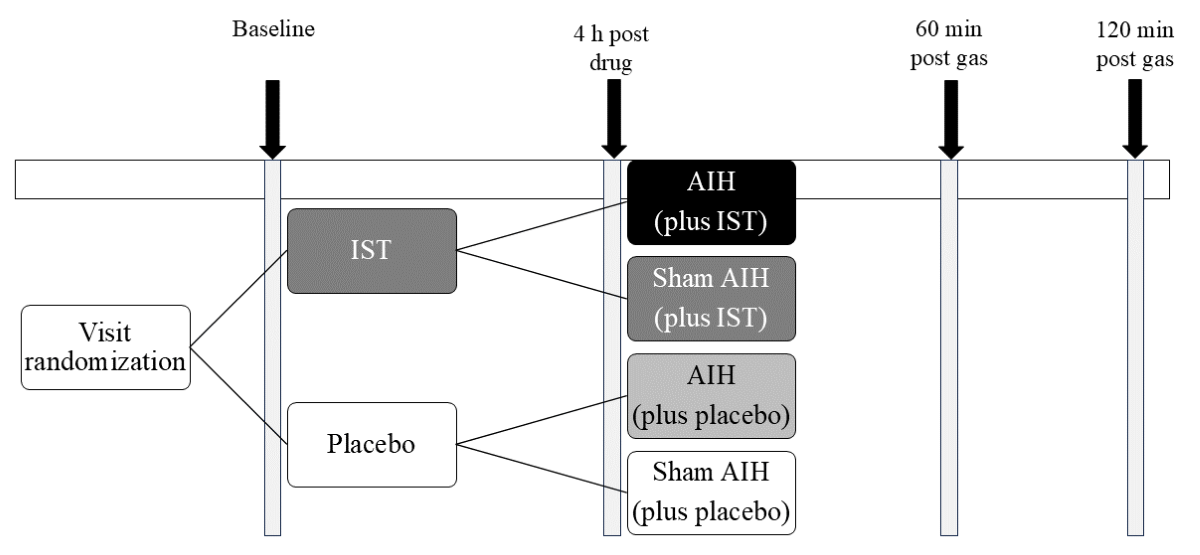
Study design. The gray bars with arrows represent the timing of data collection procedures. AIH: acute intermittent hypoxia; IST: istradefylline.

After providing written consent and completing screening, participants will complete 4 individual study visits separated by 2 weeks (±5 days). Visits will include (1) istradefylline + AIH, (2) placebo + AIH, (3) istradefylline + sham AIH, and (4) placebo + sham AIH (Figure 3), in randomized order. Participants will undergo single sessions of each treatment condition. They will be instructed to avoid exercise, caffeine, and nicotine products prior to testing for a minimum of 6 hours and to avoid eating within 1 hour of sessions.

Session order was determined by a random number table generated in advance of the enrollment of the first participant and maintained by the investigational pharmacist. Participants and investigators will remain blinded to istradefylline versus placebo for study administration and data analysis. Participants as well as the investigator collecting test outcomes will be blinded to AIH versus sham AIH. Prior to each study visit, the investigational pharmacist will inform the study coordinator which gas to administer during intervention (ie, hypoxic air for AIH vs normoxic air for sham AIH). Study team members conducting gas treatment cannot be fully blinded to AIH versus sham AIH due to monitoring of pulse oximetry oxygen saturation (SpO_2_), which is necessary for safety; thus, the investigator collecting testing outcomes will not be present during gas administration.

Participants will sit upright in a chair with the head and trunk supported while breathing through a sealed face mask. Resting ventilation, respiratory muscle activation, maximal respiratory pressures, maximal pinch force, and blood draws will be completed at the start of each study visit, then participants will receive study medication to be taken orally. After a 4-hour break, all outcome-measure testing will be repeated, followed by gas delivery. Postintervention testing will occur at 60 and 120 minutes following gas delivery. Table 2 delineates outcome measures taken at each time point.

Study visits are expected to last 7-8 hours, including equipment setup, repeated outcome measurements, oral administration of study medication, waiting periods, gas delivery, patient positioning and pressure relief, rest, and snacks or meal breaks.

**Table 2:** Timing of variable measurements throughout study visit.

	Baseline	4 h post drug	Gas delivery	60 min post gas	120 min post gas
Adverse events	✓	✓	✓	✓	✓
Vital signs (HR^a^/BP^b^)	✓	✓	✓	✓	✓
Participant-reported symptoms	✓	✓		✓	✓
Borg dyspnea scale			✓		
Laboratory tests	✓	✓		✓	
Minute ventilation	✓	✓	✓	✓	✓
MIF^c^	✓	✓		✓	
PCF^d^	✓	✓			✓
MIP^e^	✓	✓		✓	
SNIP^f^	✓	✓			✓
Pinch force	✓	✓			✓

^a^HR: heart rate.

^b^BP: blood pressure.

^c^MIP: maximal inspiratory pressure.

^d^PCF: peak cough flow.

^e^MIP: maximal inspiratory pressure.

^f^SNIP: sniff nasal inspiratory pressure.

### Independent Variable—A2A Antagonist Versus Placebo

During each study visit, participants will take a single, 20 mg NOURIANZ pill or an identical-appearing placebo. The study agent will be taken after the completion of baseline breathing tests. Study gas delivery will begin 4 hours later. During the 4-hour waiting period, participants will have a private room with a bed and recliner and access to water, snacks, and a light meal, as well as bathroom facilities. Participants will also be free to move about the research unit.

### Independent Variable—AIH Versus Sham AIH

**Figure 4:**
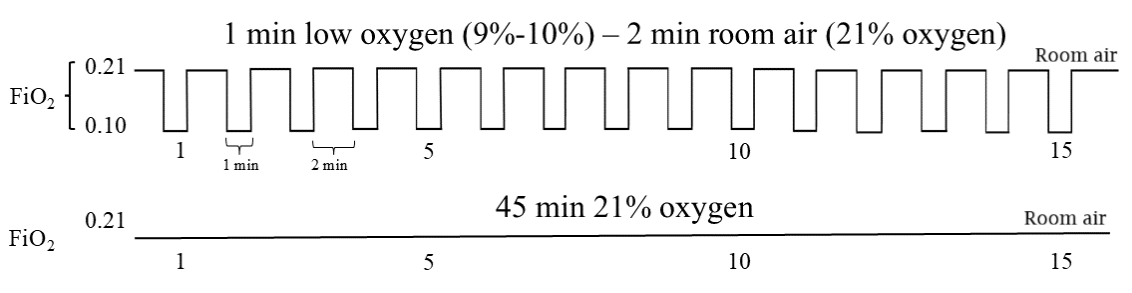
Schematic of acute intermittent hypoxia (AIH) and sham AIH interventions. AIH sessions will consist of 1-minute intervals of hypoxia (10% O2) separated by up to 2-minute intervals of normoxic room air (21% O2). Sham AIH will use identical equipment, but the gas bag will contain 21% O2.

Following the 4-hour break and second round of testing, participants will undergo a 45-minute session of either AIH (up to 1 minute of 9%-10% O_2_ with 2 minutes of 21% O_2_ for 15 cycles; target SpO_2_ nadir 82%-85%) or sham AIH (Figure 4). During sham AIH administration, the 1-minute periods of study gas will use a gas bag containing 21% O_2_, with 2-minute room-air periods, so that participants are breathing normoxic air for the entire session. Outcome measures will be repeated 60 and 120 minutes after gas delivery.

**Figure 5:**
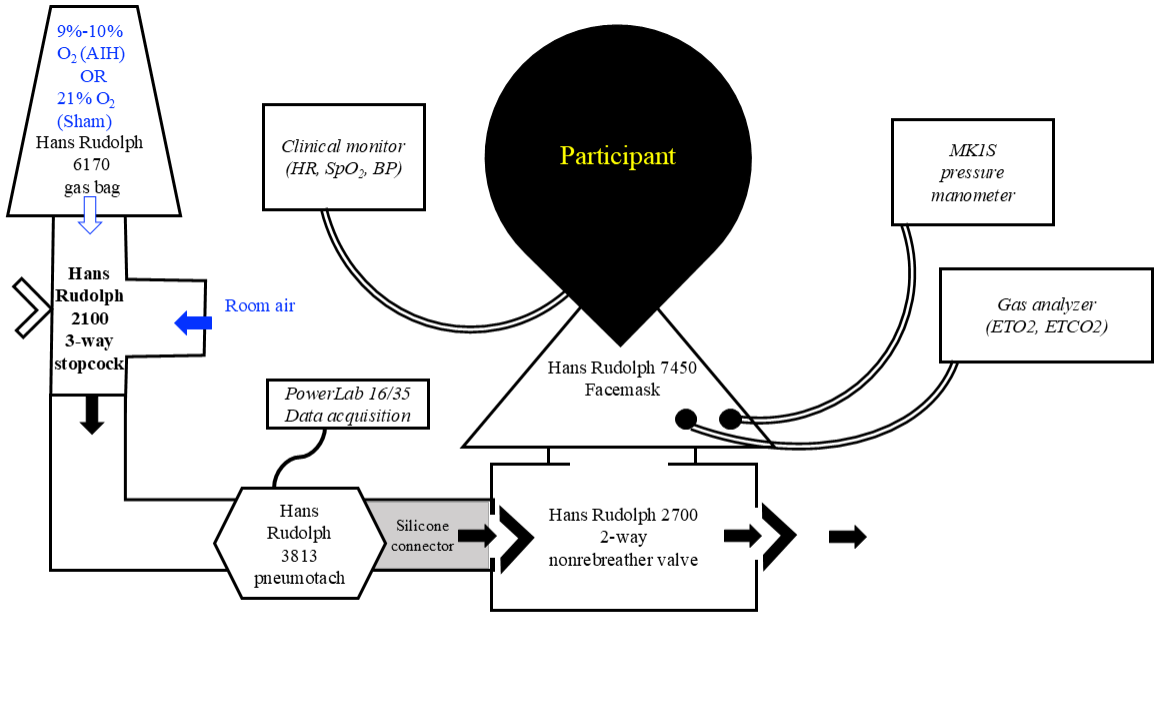
Schematic of the respiratory circuit for gas delivery. AIH: acute intermittent hypoxia; BP: blood pressure; ETCO2: end-tidal CO2; ETO2: end-tidal O2; HR: heart rate; PL: PowerLab; SpO2: pulse oximetry oxygen saturation.

A schematic of the respiratory circuit is shown in Figure 5. Participants will sit upright in a chair with the head and trunk supported while breathing through a sealed face mask (Hans Rudolph 7450). The face mask will be connected to a 2-way nonrebreathing valve (Hans Rudolph 2700) that allows the exhaled breath to escape to room air. A pressure transducer (MK1S, GM Instruments) and gas analyzer (Gemini CO_2_ & O_2_ monitor; CWE Inc) will be connected directly to the face mask connector. A pneumotachograph (Hans Rudolph 4813) will be connected to the inspiratory port of the nonrebreathing valve. Gas bags will be connected to a 3-way stopcock valve (Hans Rudolph 2100), and the direction of the valve will be manually alternated between the study gas (1-minute intervals) and normoxic room air (2-minute intervals). Respiratory data will be recorded during gas delivery, using a data acquisition system (PowerLab 16/35 and PowerLab C; ADInstruments).

Respiratory rate, targeted O_2_ value, end-tidal CO_2_, HR/rhythm, and SpO_2_ will be continuously monitored during the testing sessions. Multichannel sEMG (Trigno Centro; Delsys) will also be recorded from respiratory muscles (bilateral submental, scalene, sternocleidomastoid, second parasternal, and diaphragm) as well as abductor pollicis muscle during testing and captured in real time in LabChart (AD Instruments). At the end of each 1-minute hypoxic (or sham-hypoxic) presentation, participants will report their rating of dyspnea (10-point modified Borg scale). For every fifth presentation, noninvasive BP will be taken. We will establish an individualized communication plan (eg, hand signal, head tilt, call switch, etc) prior to study gas administration to ensure that all participants can alert the investigator of any discomfort, shortness of breath, or requests to remove the face mask.

### Safety Considerations

The target SpO_2_ during AIH is 82%-85% O_2_. If the SpO_2_ reaches the lower limit of the target range, gas delivery will be switched to the normoxic recovery time. Subsequent hypoxia durations will also be reduced by 5 seconds. AIH will be stopped in the unlikely event the HR increases or decreases >30% from the resting value, or systolic BP changes >30% from the resting value. The expected acute physiological response to hypoxia is a stable or decreased CO_2_ due to increased ventilation. However, if end-tidal CO_2_ increases >3 mmHg above baseline, we will shorten AIH bouts using the same process described above for SpO_2._ Additional stopping criteria include a respiratory rate of >30 that fails to normalize within 1 minute of normoxia, SpO_2_ <82% that fails to normalize within 1 minute of normoxia, or SpO_2_ <82% occurring within 30 seconds of hypoxia initiation.

In the unlikely event that a participant is injured as a direct result of participation in this study, professional services rendered by any UF Health Science Center health care provider will be provided without charge. These health care providers include physicians, physician assistants, nurse practitioners, dentists, and psychologists. Any other expenses, including UF Health hospital expenses, will be billed to the participant or his or her insurance provider.

Safety oversight will occur internally through the local IRB and a data safety monitoring board (DSMB). The DSMB was convened before enrollment of the first patient, after completion of the first 3 participants, and at least annually thereafter. The DSMB includes 3 members—1 expert in intermittent hypoxia, 1 clinical medicine expert who interprets or prescribes respiratory testing and treatment, and 1 rehabilitation specialist.

Testing and enrollment of participants will be paused, and DSMB will convene for any of the following events: (1) any clinically significant respiratory event, (2) a clinically significant cardiovascular event, or (3) a serious adverse event as defined by the UF IRB. Should any of the events occur, research staff shall inform the IRB and the DSMB. Any subsequent study activities would only be resumed when cleared or the protocol modified by the IRB and DSMB. As of January 2025, these serious events have not occurred, and the DSMB has only been convened for planned early and annual meetings.

### Aim 1 Dependent Variables

1. Adverse events: adverse events will be tracked and recorded per usual IRB requirements and reported as the Aim 1 primary safety outcome. It is hypothesized that there will be no difference in the rates of adverse events among study conditions.
2. Cardiopulmonary responses: vital signs and cardiac rhythm will be monitored throughout administration of each test condition. These measurements will be used in real time to monitor intervention safety, particularly during gas administration, as outlined under “safety considerations.” Additionally, 1-minute averages of HR, BP, end-tidal CO2, and SpO2 will be compared at baseline, 60 minutes, and 120 minutes following study gas delivery. It is anticipated that these measurements will remain stable at these time points.
3. Laboratory tests: baseline blood samples will be drawn for each session and include routine clinical laboratory tests (complete blood count, chemistry) and uric acid. Blood samples will be drawn for plasma istradefylline levels at the baseline of each study visit and 4 and 6 hours after ingestion of the study medication. It is anticipated that laboratory test values will remain stable across visits. Participants will be offered an intravenous line to be placed at the start of the visit to avoid multiple venipunctures.
4. Participant-reported symptoms: participants will complete 10-point visual analog scales to rate the intensity of symptoms (of dyspnea, dizziness, weakness, fatigue, nausea, and involuntary movements) at the following time points: baseline, 4 hours after taking the drug or placebo, and 60 and 120 minutes after gas delivery. It is anticipated that symptoms will remain stable across time points and visits.
5. Study completion rate: the primary feasibility outcome will be the proportion of participants who complete all study visits. Additional feasibility metrics will include screening failures, proportions of interested participants who are ineligible, and timeliness of study milestones.

### Aim 2 Dependent Variables

1. Quiet breathing assessment: the pneumotachograph and pressure transducer connected to the face mask will record breath-by-breath flow, tidal volume, airway opening pressure, and breathing rate. After achieving a stable tidal volume, minute ventilation will be recorded for 5 minutes. The primary outcome for Aim 2 is an increase in tidal volume from baseline to postintervention 120-minute follow-up. Minute ventilation will be measured at baseline, just prior to study gas delivery, and 60 and 120 minutes post gas intervention.
2. Respiratory muscle assessment: sEMGs of the respiratory muscles and the abductor pollicis muscle (nonrespiratory control muscle) will be recorded as described above to assess effects of intervention on motor control. To identify the muscles active during the quiet breathing assessment, the root mean square of each muscle signal will be averaged. Multimuscle activation will be calculated using a validated, vector-based measure that indexes the magnitude and pattern of composite electromyography activity [58]. The composite respiratory muscle activity from each participant with ALS will be contrasted to the averaged composite magnitude and pattern of the pooled control sample [59]. It is anticipated that composite magnitude will be lower in people with ALS than in controls and increase with intervention, especially with combined AIH + istradefylline.
3. MIPs: inspiratory force generation will be assessed as the maximal voluntary pressure generated by the inspiratory muscles (MIP) against a closed valve, measured at the mouth [60]. Sniff nasal inspiratory pressure measures inspiratory force generation at the nose. These tests will be used as surrogate measures of respiratory strength. Each test will be repeated until 3 measurements are obtained within 10% variability; a minimum 20-second rest will be provided between measurements. A recent systematic review reported a high level of evidence for concurrent validity for MIP; however, interrater reliability was low [61]. To minimize interrater reliability, we standardized instructions given to participants across sessions and used the same rater for each participant. MIP will be measured at baseline, immediately prior to study gas delivery (4 hours after drug administration), and 60 minutes post gas delivery. Sniff nasal inspiratory pressure will be measured at baseline, immediately prior to study gas delivery, and 120 minutes post gas delivery. We hypothesize that these measures will be lower in people with ALS than with controls and will increase with intervention, especially with combined AIH + istradefylline.
4. Maximal airflow testing: peak airflows will be collected with a pneumotachograph attached to the face mask during maximal inspiration (maximum inspiratory flow) and voluntary coughing (peak cough flow). Tests will be repeated for 3-5 trials; at least a 30-second rest will be provided between measurements, but participants may rest as long as needed. Maximum inspiratory flow will be tested at baseline, just prior to study gas delivery, and 60 minutes post intervention. Peak cough flow will be tested at baseline, just prior to study gas delivery, and 120 minutes post intervention. We hypothesize that these measures will be lower in people with ALS than with controls and will increase with intervention, especially with combined AIH + istradefylline.
5. Maximal voluntary pinch force: maximal static voluntary pinch force will be included as a nonrespiratory control. Pinch has been recognized as a sensitive marker of ALS disease severity due to preferential thenar wasting [62]. A recent study found fair to good test-retest reliability for this measure [63]. Pinch force will be evaluated in a seated position with the arm at the side in 90-degree elbow flexion and neutral forearm and wrist. The test will be repeated until 3 measurements are obtained with <10% variability, with a minimum 30-second rest between measurements. Pinch force will be measured at baseline, just prior to study gas delivery, and 120 minutes post gas intervention. We hypothesize that these measures will be lower in people with ALS than with controls and will increase with intervention, especially with combined AIH + istradefylline.

Secondary outcome measures will be interpreted as exploratory in nature.

### Sample Size, Data Management, and Statistical Analysis

The sample size calculation was based on pilot data from 8 adults with ALS showing ventilation changes in AIH versus a sham condition. The preliminary data showed 10.3% (SD 23%) larger tidal volume and 10% (SD 29%) greater minute ventilation 60 minutes post AIH, as compared to sham. Published work in rodent spinal cord injury models indicates that istradefylline paired with AIH greatly enhances improvement in diaphragm activity versus AIH or drug alone (effect size of combined approach: *d*=1.92).

Additional preliminary data using caffeine as a nonspecific adenosine receptor antagonist conservatively suggested a capacity for a 10% additional gain in ventilation. With 16 crossover participants, a repeated measures ANOVA comparing 4 conditions across 4 time points, with a pooled SD of 15.3, will be sensitive to detect a difference of power of >0.8 (*f*=0.31; *F*_3, 36_=2.86; ρ=0.5; α=.05). A target sample of 24 participants will account for potential attrition. The design includes age-matched unaffected adults as a comparator group.

Descriptive statistics, including frequencies, means, SDs, and medians, will be used when appropriate. Continuous variables will be summarized with descriptive statistics, and categorical variables will be summarized with counts and percentages. Demographics and disease-progression variables will be reported descriptively; while these are not included in a priori analyses of study aims, they may be used in an exploratory manner to interpret findings as appropriate. Change scores and percent change scores will be calculated from baseline for follow-ups at postdrug or placebo administration, 60 and 120 minutes after gas intervention. We will attempt to minimize missing data by planning the study procedures and proper biosignal acquisition, thoroughly documenting the study administration and data collection, and collecting the essential information to complete the study aims. Should missing data points still occur, statistically valid analyses that appropriately address the missing values are maximum likelihood, expectation maximization, and multiple imputation [64]. Any statistical code or algorithms developed to analyze acquired data will be available and shared upon request.

## Results

This study received funding from the ALS Association starting in January 2022 and was registered on ClinicalTrials.gov in April 2022 (Multimedia Appendix 3). Recruitment for this study began in June 2022. As of January 2025, a total of 12 people with ALS and 8 age-matched controls have completed informed consent. Of these, 1 person with ALS was withdrawn after the screening visit, and 1 was withdrawn after 2 study visits. A total of 3 of the consented potential control participants were deemed ineligible following the screening visit. Thus, 10 participants with ALS and 5 age-matched controls have completed all study procedures. Recruiting is ongoing, and the final participant will complete the study by December 2025. The publication of results is expected by the end of 2026.

## Discussion

This clinical trial will evaluate the effects of istradefylline paired with AIH on breathing function in people with ALS and unaffected age-matched controls. It is anticipated that this combined treatment will be safe and feasible, and that when combined, istradefylline and AIH will result in greater gains in respiratory function (eg, increased tidal volume and increased MIP) than either treatment alone.

Individuals living with ALS face progressive neuromuscular disease that leads to paralysis from motor neuron death. While recently approved ALS drugs offer more options to treat the disease, combinatorial strategies will likely be needed to preserve motor function and independent breathing for as long as possible. To our knowledge, this is the first clinical trial to assess the combined effects of A_2_A receptor antagonism and AIH in ALS.

AIH is easy to apply and well-tolerated by most individuals, including people with ALS in a preliminary study conducted by our group [22]. In several studies of humans with chronic spinal cord injury, similar AIH protocols did not lead to adverse events [18,65-70]. We expect AIH will, likewise, be safe and well-tolerated by people with ALS and age-matched controls in this study.

NOURIANZ (istradefylline) has been studied extensively both in participants living with PD and in healthy adult participants. Population pharmacokinetic studies were summarized previously and included both single and repeated daily administration (up to 16 weeks) of istradefylline doses ranging between 10 and 200 mg [52]. Safety was established in participants living with PD and unaffected older participants, with few and minor side effects. In controlled trials, the most common adverse events with repeated daily treatment of istradefylline included dyskinesias, dizziness, hallucinations, and insomnia. These adverse events occurred more frequently after treatment with 40 mg of istradefylline [52]. As this study will use a treatment dose of 20 mg of istradefylline, we anticipate this therapy will be safe and well tolerated by participants with ALS as well as age-matched controls.

Regarding efficacy in people with spinal cord injury, AIH also improved limb function, increased walking speed, and improved ankle strength [12,18,65,67,68,70,71]. AIH combined with task-specific training enhanced hand and walking function [65,68]. However, other studies reported variable responses, indicating that AIH efficacy can be dependent on other factors (ie, time of day, gas composition, sex, age, and genetic mutations associated with variable plasticity and rehabilitation responses) [33,72-74]. These variables require further study to optimize rehabilitation strategies for treatment efficacy.

The strengths of this study include the use of a controlled AIH protocol associated with istradefylline use and the comprehensive assessment of variables such as adverse events, vital signs, changes in resting breathing, voluntary respiratory strength, respiratory control, and maximal pinch force. Assessing these variables increases the accuracy of the results, allowing a more complete assessment of changes in response to hypoxia associated with istradefylline. The study design, a randomized, double-blind clinical trial, strengthens the validity of the findings, reducing potential bias. Recruitment of participants with early-stage ALS and random allocation to intervention and control groups ensures that the results are relevant and generalizable to this population.

This study has great importance for scientific advancement, as it investigates the applicability of AIH + istradefylline as a therapeutic intervention for individuals with ALS, a population that faces significant health management challenges. If the results show significant improvements in the studied parameters, this may indicate that AIH + istradefylline is a promising strategy to be integrated into care programs for people with ALS, complementing traditional care approaches, potentially improving the overall health of these individuals and facilitating the care process.

We acknowledge multiple limitations of this study. ALS is a heterogeneous disease, and participants may have variable disease severity, chronicity of illness, limb and bulbar involvement, and requirement of ventilatory support. This limitation will be minimized by clarifying and emphasizing the inclusion and exclusion criteria. Further, there is conflicting evidence from rodent models that A_2_A receptor activation may offer some neuroprotection, particularly in presymptomatic ALS, which raises mild concerns about its inhibition [38]. This concern is minimized in this study by the recruitment of participants with symptomatic ALS, close safety monitoring in accordance with Aim 1, and the brevity of the treatment (only 2 active doses of istradefylline). If negative effects of istradefylline are found, this possibility will need to be explored further in the future.

Additional considerations include interinvestigator variability in biosignal acquisition in sEMG and respiratory signals. To address this concern, we will train study staff to follow carefully designed and thorough standard operating procedures throughout data collection. Lastly, this study is not designed to test multiple repeated exposures of the treatment conditions and will not assess any long-term rehabilitative effects of AIH, istradefylline, or combined treatment. Rather, this study intends to inform the design of optimized therapeutic approaches and guide future studies into the rehabilitation of respiratory function in people with ALS.

## Appendix

Multimedia Appendix 1 Informed consent form for participants with amyotrophic lateral sclerosis.

Multimedia Appendix 2 Informed consent form for control participants.

Multimedia Appendix 3 Trial registration table.

Multimedia Appendix 4 Peer review report by 21CTA App Review Committee, ALS Association.

## Abbreviations

A2A: adenosine 2A receptor
AHI: apnea-hypopnea index
AIH: acute intermittent hypoxia
ALS: amyotrophic lateral sclerosis
ALSFRS-R: Amyotrophic Lateral Sclerosis Functional Rating Scale-Revised
BP: blood pressure
DSMB: data safety monitoring board
HR: heart rate
IRB: institutional review board
MIP: maximal inspiratory pressure
OSA: obstructive sleep apnea
PD: Parkinson disease
pLTF: phrenic long-term facilitation
sEMG: surface electromyography
SOD1: copper zinc superoxide dismutase 1
SPIRIT: Standard Protocol Items: Recommendations for Interventional Trials
SpO2: pulse oximetry oxygen saturation
STOP-BANG: Snoring, Tiredness, Observed Apnea, High BP-BMI, Age, Neck Circumference, and Gender
UF: University of Florida
VC: vital capacity

## Data Availability

Additional data and materials will be made available upon reasonable request to the corresponding author.
