# Supplementary material for: Acute Adenosine Receptor Antagonism in Combination With Acute Intermittent Hypoxia to Promote Breathing Plasticity in Amyotrophic Lateral Sclerosis: Protocol for a Randomized, Double-Blinded, Placebo-Controlled Trial
Source: JMIR Res Protoc. 2025 Nov 7;14:e76105. doi: 10.2196/76105 (PMC12639348; doi:10.2196/76105)
Supplement: Multimedia Appendix 1 [file resprot_v14i1e76105_app1.pdf]

UF IRB-01 Approved UF IRB-01 Approved

UF | Institutional Review Board
UNIVERSITY of FLORIDA

# *INFORMED CONSENT FORM to Participate in Research, and AUTHORIZATION to Collect, Use, and Disclose Protected Health Information (PHI)*

## INTRODUCTION

Name of person seeking your consent:___________________________________________________

Place of employment & position:_______________________________________________________

Please read this form which describes the study in some detail. A member of the research team will describe this study to you and answer all of your questions. Your participation is entirely voluntary. If you choose to participate you can change your mind at any time and withdraw from the study. You will not be penalized in any way or lose any benefits to which you would otherwise be entitled if you choose not to participate in this study or to withdraw. If you have questions about your rights as a research subject, please call the University of Florida Institutional Review Board (IRB) office at (352) 273-9600.

## GENERAL INFORMATION ABOUT THIS STUDY

**1. Name of Participant ("Study Subject")**

________________________________________________________________

**2. What is the title of this research study (this “Research Study”)?**

Acute adenosine receptor antagonism to promote breathing plasticity in ALS

**3. Whom do you call if you have questions about this Research Study (the “Study Team”)?**

Principal Investigator: Barbara K. Smith, PT, PhD (352) 294-5315 or Bksmith@ufl.edu

Study Coordinator: Julia Prascak, (352) 273-6855 or JuliaPrascak@ufl.edu

**4. Who is paying for this Research Study?**

The sponsor of this study is the ALS Association.

**5. In general, what do you need to know about this Research Study?**

Agreeing to become involved in any research is always voluntary. By signing this form, you are not waiving any of your legal rights. If you decide not to participate in this research, you will not be penalized in any way and you will not lose any benefits to which you are entitled. If you have questions

UF IRB-01 Approved UF IRB-01 Approved

about your rights as a research subject, please call the University of Florida Institutional Review Board (IRB) office at (352) 273-9600.

A description of this clinical trial will be available on http://www.ClinicalTrials.gov, as required by U.S. Law. This Web site will not include information that can identify you. At most, the Web site will include a summary of the results. You can search this Web site at any time.

**a) In general, what is the purpose of the research? How long will you be involved?**

The purpose of this research study is to determine the effects on breathing of a medication, NOURIANZ™ (istradefylline), given while breathing air with reduced oxygen for short periods of time (called acute intermittent hypoxia, or AIH). We will study breathing in people with amyotrophic lateral sclerosis (ALS). Recent studies have shown that this medication may improve function in people with other types of neuromuscular diseases. We recently completed the first study of AIH in people with ALS and healthy adults. We found that participants took deeper breaths, 60 minutes after using AIH. Importantly, participants found AIH to be comfortable and reported few, if any, side effects.

You are being asked to be in this research study because you have been diagnosed with ALS and may meet other eligibility criteria to be in the study.

You will be in this study for 1 screening visit and 4 study visits over approximately 4 weeks.
A description of this clinical trial will be available on http://www.ClinicalTrials.gov, as required by U.S. Law. This Web site will not include information that can identify you. At most, the Web site will include a summary of the results. You can search this Web site at any time.

**b) What is involved with your participation, and what are the procedures to be followed in the research?**

If you are interested in this research study, you will read and discuss this informed consent form with a member of the research staff. You will have the opportunity to read this form and ask any questions you may have prior to signing. You may also share this consent form with others that you trust, such as family members or doctors, before you decide to participate in this study.

After you sign the consent form, you will complete an in-person screening visit, plus 4 study visits over the course of about one month. Study visits will include an “AIH + istradefylline” (AIH+IST) visit, and a “sham-AIH + istradefylline” (sham+IST) visit, an “AIH + placebo (AIH+CON)” visit, and a “sham-AIH + placebo” (sham+CON) visit. If you wish, you may undergo the study screening for eligibility at the beginning of the first visit. The order of the visits will be determined by chance.

**c) What are the likely risks or discomforts to you?**

Risks associated with procedures and discomfort related to questionnaires.

**d) What are the likely benefits to you or to others from the research?**

There are no potential benefits to you for taking part in this study.

**e) What are the appropriate alternative procedures or courses of treatment, if any, that might be helpful to you?**

You will not be penalized in any way or lose any benefits if you choose not to participate in this study. It is highly recommended that you continue your regular treatment and medical follow-up with your physician.

***Additional and more detailed information is provided within the remainder of this Informed Consent form. Please read before deciding if you wish to participate in this study.***

**WHAT CAN YOU EXPECT IF YOU PARTICIPATE IN THIS STUDY?**

**6. What will be done as part of your normal clinical care (even if you did not participate in this Research Study)?**

Normal clinical care for ALS may include regular doctor visits (including visits to specialists, such as pulmonologists or neurologists), blood or urine tests, physical or occupational therapy, and respiratory care services. Your clinical care may include other services not listed here.

**7. What will be done only because you are in this Research Study?**

Each visit will be performed at the UF Clinical Research Center and the activities you will do at each visit are described below:

<u>Screening</u>

You will undergo a few screening tests to make sure you are eligible to participate in the study. You will also be asked to sign this informed consent form before participating in any activity, if you have not already done so. **Picture 1** shows the mask we use. Some people find the mask feels uncomfortable. You will have the chance to try on the mask you would use during each study visit.

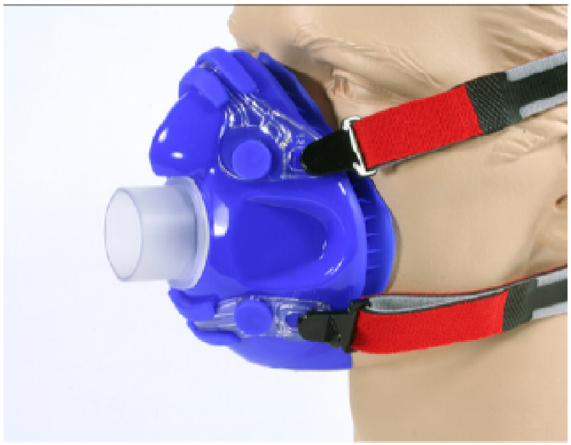

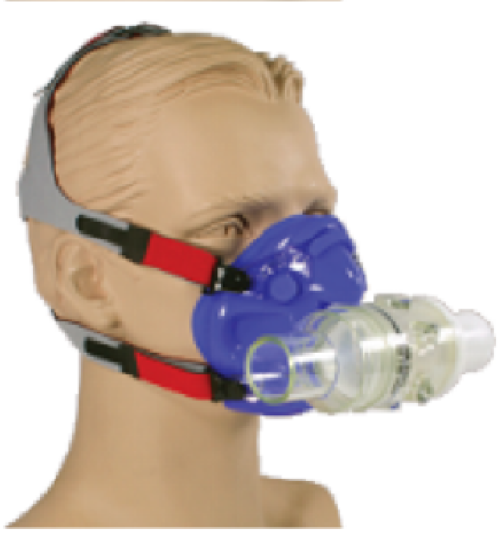

**Picture 1.** During each study visit, we use a soft, flexible mask to deliver the low or normal oxygen air.

UF IRB-01 Approved UF IRB-01 Approved

The activities for this day should take about 1-2 hours. The following tests or evaluations will be performed:

*Vital Signs, Height and Weight:* Your vital signs, height and weight will be collected.

*Urine Pregnancy Test:* If you are a female of childbearing potential, you will be asked to complete a urine pregnancy as part of the screening visit. Women who are pregnant will be ineligible to participate in the study.

*Medical History Review:* We will confirm that you have been diagnosed with ALS by reviewing your medical history. You may be asked to bring medical records to this visit to confirm your diagnosis. We will also ask you about past medical history, including the medications you currently take, that may affect your eligibility to participate in the study. You may be asked to bring your medications with you so that the study team can accurately record this information in your study record. This will take about 30 minutes.

*Vital Capacity:* This is a breathing test that measures the maximum amount of air you can breathe out in one breath. You will be asked to sit upright, take a deep breath in, and blow all of the air in your lungs into a mouthpiece. You will wear a small clip on your nose during this test, which will keep air from escaping from your nostrils. You will be asked to repeat this test a couple of times, to make sure the test is accurate. This test will take 15-20 minutes.

*12-Lead Electrocardiogram:* This is a test that measures the electrical activity of your heart. The study team will place small stickers (called electrodes) on your chest, arms, and legs and connect them to a machine that measures the electrical activity of your heart. You may be asked to remove your shirt during this test, so that the study team can place the electrodes in the right places. This test should take 5-10 minutes.

*STOP-BANG Questionnaire:* You will be asked to complete the STOP-BANG questionnaire, which is a short survey that will ask you about your breathing while you sleep. It will also ask you about your body mass index (BMI), your age, neck circumference (the measurement around your neck), and your gender. Based on the results of this questionnaire, the study team may recommend that you see a sleep specialist as part of your normal clinical care. It will be your decision if you want to visit a sleep specialist. If you decide to seek care from a sleep specialist, you will be eligible to participate in the study after you have completed 3 months of treatment. If you decide not to see a sleep specialist, you will be included in the study immediately (if you are otherwise eligible). There is no benefit to participating in the study immediately.

*Istradefylline Questionnaire:* You will be asked a few questions about current medication and supplement use, tobacco smoking status, and medical history specifically about your liver function. This will help us determine if you are eligible to participate.

*Overnight Sleep Monitor:* You will be given a sensor to take with you and wear while you sleep, to determine if you have sleep apnea (breathing that stops while you sleep). The study team will provide this device to you. The device is worn like a watch. It also has a sensor that you wear over one of your fingers, along with two stretchy sensors that you will wrap around your chest and belly. Based on the results of this test, the study team may recommend that you see a sleep specialist as part of your normal clinical care. It will be your decision if you want to visit a sleep specialist. If you decide to seek

UF IRB-01 Approved UF IRB-01 Approved

care from a sleep specialist, you will be eligible to participate in the study after you have completed 3 months of treatment. If you decide not to see a sleep specialist, you will be included in the study immediately (if you are otherwise eligible). There is no benefit to participating in the study immediately. The home sleep monitor will be ready for you to take home and wear overnight. However, if you prefer, you can choose to have us bring our equipment to your home and complete all of the screening tests there.

*Randomization:* If you decide to take part in this study and are found to be eligible during the Screening visit, you will be randomly assigned, much like the flip of a coin, as to which session you complete first. During the study you will receive AIH+CON, sham+CON, AIH+IST and sham+IST. **Picture 2** shows the events that occur during each study visit. There is no benefit to completing a specific visit first. Additionally, you will complete all four sessions listed no matter which visit you are randomized to first. You will not be told the order of the visits until the end of your fourth visit, and you will be asked about your experiences during testing. The visits will occur ~2 weeks apart (± 5 days).

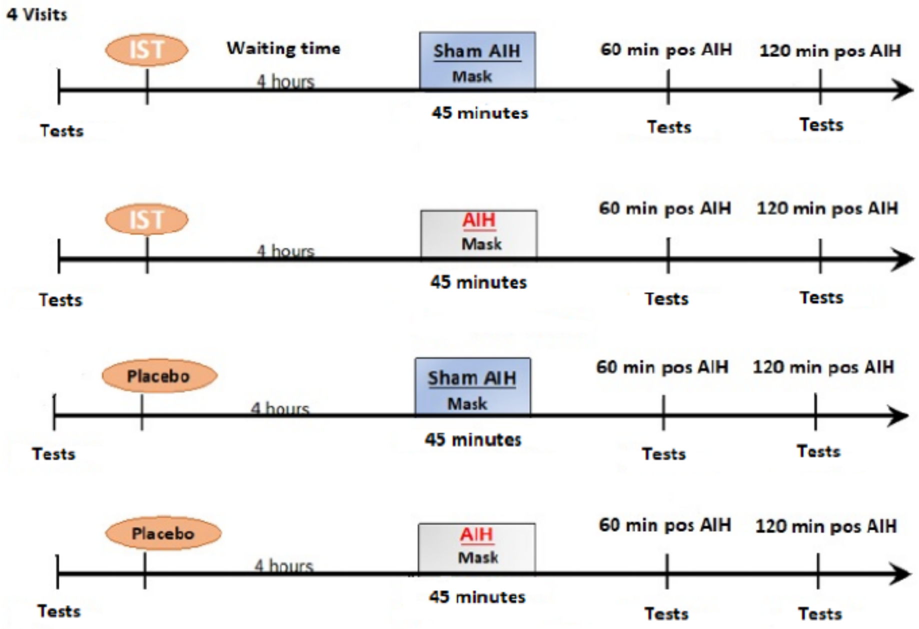


**Picture 2.** The events that occur during each study visit. The order of each visit will be determined by chance.

<u>AIH+CON Visit</u>

This visit will take approximately 7-8 hours. This visit will consist of taking a placebo medication ("sugar pill"), followed by the acute intermittent hypoxia (AIH) procedure 4 hours later. In addition we will test your breathing and strength prior to taking the control medication, and then immediately before, 60 minutes and 120 minutes after AIH. Information about each of the study tests is given in the "Study Testing" section.

We will provide a placebo medication to take by mouth 4 hours before receiving AIH. The placebo is pill about ¼-inch wide and can be taken with water. The placebo looks similar in size to the medication, but only a type of sugar. You will take the placebo at the clinical research center and will have a 4 hour waiting period in which you will be provided a private room with space to rest and/or lie down, access to

UF IRB-01 Approved UF IRB-01 Approved

water, snacks and/or light meal, and bathroom. You will be free to move about the clinical research center property until it is time for the testing.

During the AIH procedure, you will be asked to wear a mask that covers your nose and mouth. This mask will be connected to a tube and large gas-filled bag that delivers a certain amount of oxygen to you. This breathing circuit will alternate between providing normal air (which contains about 21% oxygen) and providing air with lower oxygen, which contains about 9-10% oxygen. You will receive lower oxygen for 1 minute at a time, alternating with normal air for 2 minutes, until you have reached a total of 45 minutes. During the procedure, the study staff will monitor your breathing, your heart, your pulse oximetry, and your muscle activity. You will have sensors connected to your neck, your chest, your abdomen and one of your fingers.

Sham+CON Visit

This visit will take approximately 7-8 hours. This visit will consist of taking a placebo medication ("sugar pill"), followed by the sham acute intermittent hypoxia (Sham) procedure 4 hours later. In addition we will test your breathing and strength prior to taking the control medication, and then immediately before, 60 minutes and 120 minutes after Sham-AIH. Information about each of these tests is given in the "Study Testing" section.

We will provide a placebo medication to take by mouth 4 hours before receiving AIH. The placebo is pill about ¼-inch wide and can be taken with water. The placebo looks similar in size to the medication, but only a type of sugar. You will take the placebo at the clinical research center and will have a 4 hour waiting period in which you will be provided a private room with space to rest and/or lie down, access to water, snacks and/or light meal, and bathroom. You will be free to move about the clinical research center property until it is time for the testing.

A sham procedure is a procedure that is identical in every way to the treatment being tested EXCEPT that the treatment itself is not performed. This helps researchers to determine if there is any difference between giving the treatment and doing nothing at all. During the sham procedure, you will be asked to wear the same mask that covers your nose and mouth that you will wear during the AIH procedure. It will be connected to the same breathing circuit, and you will receive normal air during the entire procedure, which will last 45 minutes. You will not alternate between breathing normal air and hypoxic air. During the procedure, the study staff will monitor your breathing, your heart, your pulse oximetry, and your muscle activity. You will have sensors connected to your neck, your chest, your abdomen and one of your fingers.

AIH+IST Visit

This visit will take approximately 7-8 hours. This visit will consist of taking istradefylline, followed by the acute intermittent hypoxia (AIH) procedure 4 hours later. In addition we will test your breathing and strength prior to taking the control medication, and then immediately before, 60 minutes and 120 minutes after AIH. Information about each of these tests is given in the "Study Testing" section.

We will provide istradefylline to take by mouth 4 hours before receiving AIH. The istradefylline is a small pill about ¼-inch wide and can be taken with water. You will take the istradefylline at the clinical research center and will have a 4 hour waiting period in which you will be provided a private room with

UF IRB-01 Approved UF IRB-01 Approved UF

space to rest and/or lie down, access to water, snacks and/or light meal, and bathroom. You will be free to move about the clinical research center property until it is time for the testing.

During the AIH procedure, you will be asked to wear a mask that covers your nose and mouth. This mask will be connected to a tube and large gas-filled bag that delivers a certain amount of oxygen to you. This breathing circuit will alternate between providing normal air (which contains about 21% oxygen) and air with a lower oxygen content, called hypoxic air (which contains about 9-10% oxygen). The air will alternate between normal air and hypoxic air every 1 to 2 minutes, until you have reached a total of 45 minutes. During the procedure, the study staff will monitor your breathing, your heart, your pulse oximetry, and your muscle activity. You will have sensors connected to your neck, your chest, your abdomen and one of your fingers.

<u>Sham+IST Visit</u>

This visit will take 7-8 hours and consist of the sham procedure, as well as strength and respiratory tests occurring immediately before, 60 minutes and 120 minutes after the procedure. Information about each of these tests is given in the “Study Testing” section.

We will provide istradefylline to take by mouth 4 hours before receiving AIH. The istradefylline is a small pill about ¼-inch wide and can be taken with water. You will take the istradefylline at the clinical research center and will have a 4 hour waiting period in which you will be provided a private room with space to rest and/or lie down, access to water, snacks and/or light meal, and bathroom. You will be free to move about the clinical research center property until it is time for the testing.

A sham procedure is a procedure that is identical in every way to the treatment being tested EXCEPT that the treatment itself is not performed. This helps researchers to determine if there is any difference between giving the treatment and doing nothing at all. During the sham procedure, you will be asked to wear the same mask that covers your nose and mouth that you will wear during the AIH procedure. It will be connected to the same breathing circuit, and you will receive normal air during the entire procedure, which will last 45 minutes. You will not alternate between breathing normal air and hypoxic air. During the procedure, the study staff will monitor your breathing, your heart, your pulse oximetry, and your muscle activity. You will have sensors connected to your neck, your chest, your abdomen and one of your fingers.

<u>Study Testing</u>

At each of the 4 study visits: AIH+CON, sham+CON, AIH+IST, and sham+IST, you will undergo a blood draw three times: upon arriving to the testing center, immediately before receiving AIH or sham, and 120 minutes after the AIH or sham procedure. Your blood will be tested to measure istradefylline levels, uric acid (a compound in your blood), and specific markers related to the body’s response to istradefylline and AIH.

In addition, you will perform the following tests at the start of each visit, and then immediately before, 60 minutes and 120 minutes after the AIH or sham procedure:

*Questionnaires about your symptoms:* Several times during your study visit, we will ask you to rate whether you have several symptoms. These symptoms include: shortness of breath, nausea, involuntary movements, dizziness, and fatigue. Will ask you about these symptoms at the start of each visit, and

UF IRB-01 Approved UF UF IRB-01 Approved

then immediately before, 60 minutes and 120 minutes after the AIH or sham procedure. We will also ask about any shortness of breath several times during each AIH or sham procedure.

*Vital signs:* We will record your heart rate, breathing rate and pattern, blood pressure, and oxygen and carbon dioxide gas levels, at the start of each visit, and then immediately before, 60 minutes and 120 minutes after the AIH or sham procedure. We will also measure your heart rate and oxygen throughout each AIH or sham procedure, and take your blood pressure several times.

*Resting breathing:* While wearing a facemask, you will breathe normally for 5 minutes. The study team will measure your breathing in several ways, including your breathing rate and how much air you take with each breath. This test will take about 5 minutes.

*Surface EMG:* You will have sensors placed on your neck, chest and abdomen to measure the activity of your muscles. These same sensors will be used during the AIH, sham, AIH+IST and SHAM+IST procedures. The study team will collect these measurements as you complete the other tests, and it will not take additional time.

*Respiratory Pressure Testing:* These tests measure the tension you can create with your breathing muscles. The tests are done while sitting in a chair. We will use a facemask or mouthpiece, along with a nasal sensor, to measure the amount of tension you make while breathing/sucking/sniffing as deeply as possible. These tests may be repeated until the study team determines that the measurements are accurate. These tests take about 5-10 minutes.

*Airflow and Cough Testing:* This test measures how quickly you inhale air and the force of your cough. The test is done while you are seated on a chair. While wearing the facemask, you will be asked to breathe in deeply and cough as hard as you can with a few seconds of rest in-between the coughs. We will ask you to repeat the test 2-3 times. During the test, we will measure the flow of air as you breathe in and cough out, along with your breathing pattern. This test takes 2-4 minutes.

*Pinch Strength:* While seated, you will be asked to squeeze a sensor between your thumb and index finger as hard as you can for ~5 seconds. The sensor will measure your pinch strength. This test may be repeated until the study team determines that the measurements are accurate. This test will take about 3-5 minutes.

*Overnight capnography test:* A capnography test measures the level of carbon dioxide in your blood, through a sensor worn on the skin. The breathing muscles sometimes work less efficiently during sleep, and capnography sensor detects how frequently this happens. This is an optional test that will occur once during your participation. If you elect to wear the skin sensor, we will loan you with the equipment and sensor to take home between study visits.

Tests done only for research purposes will not be evaluated or used to diagnose or treat any of your medical problems. These may need to be repeated if required for your medical care in the future.

Once this research study is completed, the information could be used for future research studies or distributed to another investigator for future research studies without additional informed consent from you or your legally authorized representative.

UF IRB-01 Approved UF IRB-01 Approved

If any identifiable information or biospecimens were collected as part of this research, it is possible that your research information or specimens, with all personally identifiable information removed, could be used for future research studies or distributed to another investigator for future research studies without additional informed consent from you or your legally authorized representative.

If you have any questions now or at any time during this Research Study, please contact one of the Research Team members listed in question 3 of this form.

**8. What identifiable health information will be collected about you and how will it be used?**

Your protected health information may be collected, used, and shared with others to determine if you can participate in the study, and then as part of your participation in the study. This information can be gathered from you or your past, current or future health records, from procedures such as physical examinations, x-rays, blood or urine tests or from other procedures or tests. This information will be created by receiving study treatments or participating in study procedures, or from your study visits and telephone calls. More specifically, the following information may be collected, used, and shared with others:

- Your medical history, including past conditions and surgeries, medications you take or have taken, and information related to your diagnosis of ALS
- Your social security number, for reimbursement purposes
- Demographic information
- Contact information, such as phone number and address
- Results of the research tests done for this study

The Research Team may collect this information from other healthcare providers, such as laboratories, which are a part of this research, as well as healthcare providers that are not part of this research (other doctors, hospitals or clinics). Other professionals at the University of Florida or Shands Hospital who provide study-related care, and the University of Florida Institutional Review Board (IRB), may also collect your health information.

The Research Team listed in question 3 above will use or share your health information as described below to carry out this research study.

**9. With whom will this health information be shared?**

This health information may be shared with:

- the study sponsor (listed in Question 4 of this form);
- other professionals at the University of Florida or Shands Hospital that provide study-related treatment or procedures.
- United States governmental agencies which are responsible for overseeing research, such as the Food and Drug Administration, the Department of Health and Human Services, and the Office of Human Research Protections;
- government agencies which are responsible for overseeing public health concerns, such as the Centers for Disease Control and federal, state and local health departments,
- the IRB that reviewed this Research Study and ensures your rights as a Study Subject are protected.

Otherwise, your identifiable health information will not be shared without your permission unless required by law or a court order. Once your health information is shared with those listed above, it is possible that they could share it without your permission because it would no longer be protected by the federal privacy law.

**10. How long will you be in this Research Study?**

Your PHI will be used and shared with others for at least three years beyond the end of the study to allow time to analyze the data and prepare any publications that result from the study. De-identified study data (information that cannot be traced to you) will be kept indefinitely in a database so that we can use it for comparisons in the future.
You are not required to sign this consent and authorization or allow researchers to collect, use and share your PHI. Your refusal to sign will not affect your treatment, payment, enrollment, or eligibility for any benefits outside this research study. However, you cannot participate in this research unless you allow the collection, use and sharing of your protected health information by signing this consent and authorization.

You have the right to review and copy your protected health information. However, we can make this available only after the study is finished.

You can revoke your authorization at any time before, during, or after your participation in this study. If you revoke it, no new information will be collected about you. However, information that was already collected may still be used and shared with others if the researchers have relied on it to complete the research. You can revoke your authorization by giving a written request with your signature on it to the Principal Investigator.

This Authorization to use and share your health information expires at the end of the study, unless you revoke it (take it back) sooner.

**11. How many people are expected to take part in this Research Study?**

We will enter up to 40 people into this study, to have 24 ALS patients and 16 healthy adults complete this study.

**WHAT ARE THE RISKS AND BENEFITS OF THIS STUDY AND WHAT ARE YOUR OPTIONS?**

**12. What are the possible discomforts and risks from taking part in this Research Study?**

Risks associated with the AIH procedure: Although previous studies have shown that it is hard for subjects to tell when they are breathing hypoxic air for short periods of time, it is possible this procedure will make you feel short of breath. You may feel tired or lightheaded. The sensors and face mask attached to you during the procedure may also be uncomfortable. Wearing the face mask may feel warm and moist. However, we provide a fan to keep you cool. The breathing circuit can be attached as needed to provide a break. The other testing sensors contain tape that may feel uncomfortable or itchy. Your breathing and pulse oximetry (a measurement of how much oxygen is getting to your body) will be monitored throughout the procedure, and the procedure will be stopped if the study team decides it is best for you. You can also stop the procedure at any time.

UF IRB-01 Approved UF IRB-01 Approved

Risks associated with the sham procedure: The sensors, facemask and nose clip may feel uncomfortable. You can also stop the procedure at any time. Wearing the face mask may feel warm and moist. However, we provide a fan to keep you cool. The breathing circuit can be attached as needed to provide a break. The other testing sensors contain tape that may feel uncomfortable or itchy.

Risks associated with istradefylline consumption: Some people taking istradefylline have reported one or more of the following side effects: uncontrolled sudden movements, low blood pressure when standing, dizziness, hallucination (feeling sensations that are not real), and trouble sleeping. Some people also report stomach problems, such as nausea, vomiting, intestinal problems, decreased desire to eat, and diarrhea. These side effects were seen more frequently in people who took higher doses of istradefylline (40 mg) than the dose we use (20 mg), and in patients with Parkinson's Disease (PD) who took other PD medications at the same time. If any of these side effects occur, please notify a member of the study team.

Risks associated with blood draws: The risks of drawing blood from a vein include discomfort at the site of puncture; possible bruising and swelling around the puncture site; rarely an infection; and, uncommonly, faintness from the procedure.

Risks associated with breathing tests: Although each breathing test lasts for a very short time, the testing may feel strenuous. The breathing tests are associated with a very low risk of difficulty, even for sick patients hospitalized with a breathing machine. Your breathing and heart function will be monitored during the testing sessions. You can stop any test at any time, and you will be provided with plenty of rest, whether you feel tired or not.

Risks associated with pinch strength testing: Your hand may feel tired following this procedure. This test lasts a very short time and you will be able to rest afterward.

Risks associated with ECG, surface EMG and overnight sleep monitoring: The sensors attached to you during these tests may be uncomfortable and can occasionally cause mild skin irritation.

Risks associated with questionnaires: Questionnaires may ask you some questions that may be uncomfortable to answer. You can skip a question at any time and still participate in the study.

Researchers will take appropriate steps to protect any information they collect about you. However, there is a slight risk that information about you could be revealed inappropriately or accidentally. Depending on the nature of the information, such a release could upset or embarrass you, or possibly affect your insurability or employability. Questions 17-21 in this form discuss what information about you will be collected, used, protected, and shared.

This Research Study may also include risks that are unknown at this time.

Please note, participating in more than one research study or project may further increase the risks to you. If you are already enrolled in a research study, please inform one of the Research Team members listed in question 3 of this form or the person reviewing this consent with you before enrolling in this or any other research study or project.

During the study, the Research Team will notify you of new information that may become available and might affect your decision to remain in the study.

UF IRB-01 Approved UF IRB-01 Approved

The University of Florida is required by law to protect your health information. Your health information will be stored in locked filing cabinets or on computer servers with secure passwords, or encrypted electronic storage devices, as required by University policy. However, there is a slight risk that information about you could be released inappropriately or accidentally. Depending on the type of information, a release could upset or embarrass you, or possibly affect your ability to get insurance or a job.

If you wish to discuss the information above or any discomforts you may experience, please ask questions now or call one of the Research Team members listed in question 3 in this form.

**13a. What are the potential benefits to you for taking part in this Research Study?**

There are no potential benefits to you for taking part in this study.

**13b. How could others possibly benefit from this Research Study?**

Information gained through this research study could help researchers find better ways to treat people with ALS and other similar neuromuscular diseases.

**13c. How could the Research Team members benefit from this Research Study?**

In general, presenting research results helps the career of a researcher. Therefore, the Research Team listed in question 3 of this form may benefit if the results of this Research Study are presented at scientific meetings or in scientific journals.

**13d. Will you be allowed to see the research information collected about you for this Research Study?**

You may not be allowed to see the research information collected about you for this Research Study, including the research information in your medical record, until after the study is completed. When this Research Study is over, you will be allowed to see any research information collected and placed in your medical record.

**14. What other choices do you have if you do not want to be in this study?**

You are not required to be in this study. Your other option is to continue with your normal clinical care.

You may also refuse to authorize the use of your health information, but if you refuse, you may not be allowed to be in this research study or receive any research-related treatment that is only available in this research study. However, your decision not to sign this Authorization will not affect any other treatment you may be eligible to receive.

**15a. Can you withdraw from this study?**

You may withdraw your consent and stop participating in this Research Study at any time. If you do withdraw your consent, there will be no penalty to you, and you will not lose any benefits to which you are otherwise entitled. If you decide to withdraw your consent to participate in this Research Study for

UF IRB-01 Approved UF IRB-01 Approved

any reason, please contact the Research Team listed in question 3 of this form. They will tell you how to safely stop your participation.

You can also change your mind and take back this Authorization at any time by sending a written notice to the Research Team listed in question 3 of this form to let them know your decision. If you take back this Authorization, the Research Team may only use and disclose your health information already collected for this research study. No additional health information about you will be collected or disclosed to the Research Team. However, if you take back this Authorization, you may not be able to continue in this study. Please discuss this with a member of the Research Team listed in question #3.

**15b. Can the Principal Investigator withdraw you from this Research Study?**

You may be withdrawn from this Research Study without your consent for the following reasons:

- The researchers determine that you are unable to complete the tests and procedures required for the study.
- The individuals who oversee the study decide to stop the study.
- The researchers determine that it is in your best interest to withdraw you from the study.

## WHAT ARE THE FINANCIAL ISSUES IF YOU PARTICIPATE?

**16. If you choose to take part in this Research Study, will it cost you anything?**

It will not cost you anything to participate in this study.

**17. Will you be paid for taking part in this Research Study?**

Yes, you will be paid $20 for completing the screening visit and $120 for each of the four sessions: AIH+CON, sham+CON, AIH+IST, sham+IST, and you will be reimbursed up to $250 for your travel expenses for each study visit. If you decide to withdraw from the study before you complete all of the visits, you will be reimbursed for the visits you completed.

Your payment for participation in this research study is handled through the University of Florida's Research Participant Payments (RPP) Program. Your information which will include your name, address, date of birth, and SSN (depending on amount of money you are paid) is protected. Access to the (RPP) Program site is limited to certain staff with the assigned security role. You will be randomly assigned a specific identification (ID) number to protect your identity.
If you have any problems regarding your payment contact the study coordinator.

If you are paid more than $199 for taking part in this study, your name and social security number will be reported to the appropriate University employees for purposes of making and recording the payment as required by law. You are responsible for paying income taxes on any payments provided by the study. Payments to **nonresident aliens** must be processed through the University of Florida Payroll and Tax Services department. If the payments total $600 or more in a calendar year, the University must report the amount you received to the Internal Revenue Service (IRS). The IRS is not provided with the study name or its purpose. If you have questions about the collection and use of your Social Security Number, please visit: http://privacy.ufl.edu/SSNPrivacy.html.

UF IRB-01 Approved UF IRB-01 Approved

**18. What if you are injured while in this Research Study?**

If you are injured as a direct result of your participation in this study, only the professional services that you receive from any University of Florida Health Science Center healthcare provider will be provided without charge. These healthcare providers include physicians, physician assistants, nurse practitioners, dentists or psychologists. Any other expenses, including Shands hospital expenses, will be billed to you or your insurance provider.

You will be responsible for any deductible, co-insurance, or co-payments. Some insurance companies may not cover costs associated with research studies or research-related injuries. Please contact your insurance company for additional information.

The Principal Investigator will determine whether your injury is related to your participation in this study.

No additional compensation is routinely offered. The Principal Investigator and others involved in this study may be University of Florida employees. As employees of the University, they are protected under state law, which limits financial recovery for negligence.

Please contact one of the Research Team members listed in question 3 of this form if you experience an injury or have questions about any discomforts that you experience while participating in this Research Study.

UF IRB-01 Approved UF IRB-01 Approved UF

## SIGNATURES

As an investigator or the investigator's representative, I have explained to the participant the purpose, the procedures, the possible benefits, and the risks of this Research Study; the alternative to being in the study; and how the participant's protected health information will be collected, used, and shared with others:

________________________________________ ____________________

Signature of Person Obtaining Consent and Authorization Date

You have been informed about this study's purpose, procedures, possible benefits, and risks; the alternatives to being in the study; and how your protected health information will be collected, used and shared with others. You have received a copy of this form. You have been given the opportunity to ask questions before you sign, and you have been told that you can ask questions at any time.

You voluntarily agree to participate in this study. You hereby authorize the collection, use and sharing of your protected health information as described above. By signing this form, you are not waiving any of your legal rights.

________________________________________ ____________________

Signature of Person Consenting and Authorizing Date
