# Supplementary material for: Acute Adenosine Receptor Antagonism in Combination With Acute Intermittent Hypoxia to Promote Breathing Plasticity in Amyotrophic Lateral Sclerosis: Protocol for a Randomized, Double-Blinded, Placebo-Controlled Trial
Source: JMIR Res Protoc. 2025 Nov 7;14:e76105. doi: 10.2196/76105 (PMC12639348; doi:10.2196/76105)
Supplement: Multimedia Appendix 3 [file resprot_v14i1e76105_app3.pdf]

Multimedia Appendix 1 Trial registration data

Data category	Information
Primary registry and trial identifying number	ClinicalTrials.gov NCT05377424
Date of registration in primary registry	26 April 2022
Secondary identifying numbers	IRB202101568
Source(s) of monetary or material support	ALS Association
Primary sponsor	ALS Association
Secondary sponsor(s)	NA
Contact for public queries	bksmith@ufl.edu
Contact for scientific queries	Barbara Smith, DPT, PhD, Physical Therapy Department, University of Florida
Public title	Acute Adenosine Receptor Antagonism to Promote Breathing Plasticity in ALS
Scientific title	Adenosine 2A Receptor Antagonism and AIH in ALS
Countries of recruitment	United States
Health condition(s) or problem(s) studied	Breathing in ALS
Intervention(s)	Experimental: AIH + istradefylline (AIH+IST)<br>During this study arm, participants will ingest a 20mg tablet containing istradefylline. Four hours later, participants will receive acute intermittent hypoxia (AIH). Breathing and pinch strength will be tested prior to taking the medication, and then immediately before, 60 minutes and 120 minutes after AIH. Participants will breathe 15 episodes/session of acute low oxygen. Air concentrations will be monitored to ensure delivery of 1-minute episodes of low oxygen, with 2 minutes room-air intervals. Respiratory rate, oxygen saturation, heart rate/rhythm, and blood pressure will be monitored throughout the session.
	Active Comparator: Sham-AIH + istradefylline (sham+IST)<br>This is a sham counterpart to the low oxygen. During this study arm, participants will ingest a 20mg tablet containing istradefylline. Four hours later, participants will receive sham acute intermittent hypoxia (sham). Breathing and pinch

Data category	Information
	strength will be tested prior to taking the medication, and then immediately before, 60 minutes and 120 minutes after sham. Participants will breathe 15 episodes/sessions of sham low oxygen, in which normal air is used. One-minute episodes of sham low oxygen are separated by 2 minutes room-air intervals. Respiratory rate, oxygen saturation, heart rate/rhythm, and blood pressure will be monitored throughout the session.
	Active Comparator: AIH + placebo (AIH+CON)<br>This is a placebo counterpart to the istradefylline drug. During this study arm, participants will ingest a 20mg tablet containing microcrystalline cellulose. Four hours later, participants will receive acute intermittent hypoxia (AIH). Breathing and pinch strength will be tested prior to taking the medication, and then immediately before, 60 minutes and 120 minutes after AIH. Participants will breathe 15 episodes/session of acute low oxygen. Air concentrations will be monitored to ensure delivery of 1-minute episodes of low oxygen, with 2 minutes room-air intervals. Respiratory rate, oxygen saturation, heart rate/rhythm, and blood pressure will be monitored throughout the session.
	Active Comparator: Sham-AIH + placebo (sham+CON)<br>This is a sham counterpart to low oxygen, and a placebo counterpart to the istradefylline drug. During this study arm, participants will ingest a 20mg tablet containing microcrystalline cellulose. Four hours later, participants will receive sham acute intermittent hypoxia (sham). Breathing and pinch strength will be tested prior to taking the medication, and then immediately before, 60 minutes and 120 minutes after sham. Participants will breathe 15 episodes/sessions of sham low oxygen, in which normal air is used. One-minute episodes of sham low oxygen are separated by 2 minutes room-air intervals. Respiratory rate, oxygen saturation, heart rate/rhythm, and blood pressure will be monitored throughout the session.
Key inclusion and exclusion criteria	Ages eligible for study: 21 Years to 80 Years; Sexes eligible for study: both; Accepts healthy volunteers: yes
	Inclusion Criteria:<br>Non-smoking adults aged 21-80 years will be eligible to participate.<br>Upon screening, eligible patients will have an ALS diagnosis (El Escorial diagnostic classifications of probable/definite ALS), vital capacity (VC) > 60% of predicted value, and ALS Functional Rating Scale (ALSFRS-R) scores of 2 or greater for bulbar and respiratory items: swallowing, speech, salivation, dyspnea, orthopnea, and respiratory insufficiency. Additionally, patients taking riluzole and/or edaravone must be on a stable dose for >30 days. Unaffected control subjects will be eligible if they have a vital capacity (VC) > 60% of predicted value.
	Exclusion Criteria:<br>Patient and controls are ineligible if they: are pregnant, have an active respiratory infection, took antibiotics within 4 weeks, are diagnosed with another neurodegenerative disease, have symptomatic cardiovascular disease or dysrhythmias (resting tachycardia and hypertension), exhibit history or presence of hypoxemia or hypercapnia, presence of rest tachypnea (RR >30), have a BMI

Data category	Information
	>35 kg/m2, have a seizure disorder, take respiratory inhalers daily for airway disease, or require external respiratory support while awake and upright, or supplemental oxygen at rest or at night.<br>In addition, the following conditions are exclusionary for the use of istradefylline: routine use of CYP3A4 inducers (i.e. carbamazepine, phenobarbitol, rifampin, phenytoin, St. John's Wort, glucocorticoids) or medications that may suppress ventilation, history of moderate renal impairment or severe hepatic impairment, and history of hallucinations or psychosis. Patients who cannot safely swallow thin liquids (required for administration of istradefylline and placebo) will also be ineligible.
Study type	This repeated measures, placebo-controlled, randomized study will study feasibility and efficacy of istradefylline, an adenosine 2A receptor antagonist in conjunction with acute intermittent hypoxia (AIH).<br>Participation in this study includes a screening for eligibility, plus 4 individual study visits separated by 1 week. The eligibility screening will include a review of medical history and medications, along with a breathing test and sleep study. Each participant will experience a different study condition on each of their 4 study visits: an "AIH + istradefylline" (AIH+IST) visit, and a "sham-AIH + istradefylline" (sham+IST) visit, an "AIH + placebo (AIH+CON)" visit, and a "sham-AIH + placebo" (sham+CON) visit. The visits will be in random order for each subject. Participants and the testing investigators will not be told which order the visits will be. Participants need to avoid exercise and caffeine and nicotine products for >6 hours before each study visit.<br>The study will assess vital signs, patient-reported symptoms, resting breathing, strength of the breathing muscles, and maximal voluntary pinch force at the start of each visit. These measures will then be repeated 1 and 2 hours after AIH or sham. Throughout the AIH and sham interventions, respiratory rate, oxygen saturation, end-tidal carbon dioxide (CO_2), heart rate, and blood pressure will be monitored.<br>For the primary efficacy endpoint, the study will measure breath volume at the start of each visit, and 1 and 2 hours after the AIH and sham interventions. A linear mixed model will be used to compare differences in tidal volume. Main effects include treatment and time, with participants as random effects.
Date of first enrolment	June 2022
Target sample size	Enroll 40 to complete 32
Recruitment status	Recruiting
Primary outcome(s)	Treatment differences in the rate of adverse events. [Time Frame: Through study completion (an average of 4-6 weeks)]<br>- Any reported adverse events will be tracked and recorded.<br>Change in resting tidal volume [Time Frame: 120 minutes after AIH]<br>-Averaged volume of breaths at rest
Key secondary outcomes	Plasma Istradefylline [Time Frame: 4 hours post- istradefylline or placebo]<br>- Blood test to measure change in level of istradefylline

Data category	Information
	Plasma Istradefylline [Time Frame: 6 hours post- istradefylline or placebo]<br>- Blood test to measure change in level of istradefylline<br>Subject-reported involuntary movements [Time Frame: 4 hours post-istradefylline or placebo]<br>- Participants will use a 0-10 scale to report the intensity of any involuntary movements or tremors. (a higher number would correspond to more involuntary movements or tremors)<br>Change in minute ventilation [Time Frame: 120 minutes post-intervention.]<br>- Change in the average volume of air during a minute of resting breathing.
