# Supplementary material for: Acute Adenosine Receptor Antagonism in Combination With Acute Intermittent Hypoxia to Promote Breathing Plasticity in Amyotrophic Lateral Sclerosis: Protocol for a Randomized, Double-Blinded, Placebo-Controlled Trial
Source: JMIR Res Protoc. 2025 Nov 7;14:e76105. doi: 10.2196/76105 (PMC12639348; doi:10.2196/76105)
Supplement: Multimedia Appendix 4 [file resprot_v14i1e76105_app4.pdf]

Applicant: Smith, Barbara
Title: Acute adenosine receptor antagonism to promote breathing plasticity in ALS
Program: Clinical Trial Awards
Institution: University of Florida
App #: 904040

View Review Information

Close Window Print

Committee: 21CTA App review

Reviewer Role: Reviewer (144775)

SUMMARY OF GRANT PROPOSAL

Please provide a brief synopsis of the clinical trial and the overall goals of the proposal.:

This Phase I-II study is designed to examine the acute effects of istradefylline- a A2AR antagonist -on respiratory function in ALS patients.
the PI proposes to to pair a single dose of istradefylline with acute intermittent hypoxia (AIH), which stimulates respiratory plasticity

Given that A2AR antagonism attenuates the death of phrenic motor neurons and that AIH increases breathing in ALS patients, the study proposes to investigate a therapeutic strategy that uses the combination of istradefylline and AIH to facilitate breathing in ALS patients. The ultimate goal is to preserve respiratory function and-therefore- independent breathing as long as possible.

This is a double-blinded, randomized, placebo-controlled study performed at a single site (University of Florida) that will include a patient cohort of 24 adults diagnosed with ALS and 16 sex and age-matched controls.
The protocol will use a single-dose (20 mg) of istradefyllin with repeated measures: a screening visit and 4 separate study visits once a week.
Each study subject will undergo 4 different conditions: (1) 1) istradefylline + AIH, (2) istradefylline + sham hypoxia, (3) placebo + AIH, and (4) placebo + sham hypoxia- with the order of these conditions randomized. at each At each visit, the following parameters will be measured at the onset of visit: Resting ventilation, respiratory muscle activation, maximal

respiratory pressures, maximal grip force, and blood. Then, patients will be given istradefyllin (or placebo) and 4 hrs after administration of istradefyllin (time to reach peak levels), the same parameters will be evaluated again, and AIH (or sham hypoxia) performed for 45 minutes. Outcome measures repeated again at 5-6 hrs. With the study visits 1 week apart to ensure drug wash-out, participation is expected to be completed in less than 6 weeks. the entire study is expected to last 24 months.

BIOLOGY

Please comment on the rationale/choice for the drug target/pathway and how relevant and important you think it is in ALS. Is there sufficient rationale provided for the biological links of the target to the disease?:

The study is based on the rationale that adenosinergic pathology has been observed in ALS and that accumulation of extracellular adenosine has been shown to enhance toxicity in C9orf72 as well as sporadic ALS. It focuses on A2AR receptors for the following reasons: (1) contrary to A1AR, which are scarce in motor neuron, A2ARs are abundant in motor neurons- especially in phrenic motor neurons that are highly vulnerable. Additionally, studies primarily done in rodent models of disease, have shown increased A2AR expression and more recently selective A2AR upregulation has also been detected in post-mortem spinal cord tissue of ALS patients.

Finally, while the A1AR is often associated with neuroprotective actions, A2ARs are associated to enhancement of excitotoxicity, since these receptors facilitate glutamate release and inhibit glutamate uptake- which we know are critical in ALS. Thus, the idea is that selective inhibition of A2AR, may reduce toxicity and delay motor neuron death in phrenic motor neurons.

Despite the evidence above and cited by the PIs that would support inhibition of A2AR, there are also studies pointing toward a beneficial role of A2A receptors (A2AR), most probably at early disease states, and a detrimental role of caffeine-mediated A2AR inhibition, in clear contrast with what occurs in other neurodegenerative diseases. Because of the studies showing positive effects of A2AR activation in prolonging facilitation in phrenic nerve activity, A2AR stimulation, contrary to inhibition, has been proposed as a potentially useful approach to increase phrenic motor neuron excitability in conditions such as spinal cord.

Unfortunately, this proposal does not address this contrasting literature and, therefore, does not consider the possible deleterious effects of A2AR inhibition, slightly reducing the enthusiasm for an otherwise quite interesting study and approach.

However, as largely discussed in the literature, these differences in function and toxicity of the A2ARs in ALS may be due to the fact that there are time windows in disease where A2AR prove beneficial and others where their blockade is required (most likely early on in disease). In line with this and until mechanistic studies show precisely how adenosine receptors are involved in motor neuron dysfunction and whether the early changes in purinergic signaling are compensatory or triggers of disease, the studies proposed here have merit. First, because there is support for inhibiting A2AR to reduce exitotoxicity. Second, because the dose of istradefyllin used here is safe and seems to be well tolerated. And third, because the study will not use a chronic administration of the drug. This is a proof-of-principle study that proposes an interesting approach that may be a useful for a limited amount of time (early stages) until those nerve cell succumb to degeneration.

The combination with AIH to promote plasticity is also quite interesting and AIH seems to be working in pilot studies performed by the PI.

There is solid rationale to support the use of AIH which we know strengthens spinal synaptic pathways to spared/still functional motor neurons, producing respiratory motor facilitation via serotonin-dependent signaling mechanisms.

STRENGTH OF PRECLINICAL OR CLINICAL DATA

Please comment on the preclinical package provided for the therapeutic. Do the preclinical data on pharmacology, efficacy, PK/PD, safety/toxicology, etc. justify moving the therapeutic to the clinical stage? If the therapeutic has been tested in the clinic before, are there adequate safety, PK, target engagement data that supports further clinical development of this drug.:

Most of the preclinical and clinical data come from other diseases. Istradefylline is commercially available as. Nourianz and approved and approved as adjunctive therapy to decrease “off time,” in adults with Parkinson’s disease (PD). So, although not in pre-clinical models of ALS, pharmacokinetic and pharmacodynamic as well as target engagement studies, have been done for this drug.

The drug is safe- at least in other neurological disease patients- at the 20 mg dose used here.

AIH has been shown to facilitate respiratory motor function in ALS animal models as well as in chronic spinal cord injury patients. In the SOD1G93A transgenic rats , even a single AIH session has been shown to enhance respiratory function.

There are no published studies showing safety and efficacy of the two strategies combined (Istradefylline + AIH), but the PI has performed preliminary tests in few patients showing feasibility-and presumably safety-of the combination at least in healthy individuals. The combination also seems effective as it appears that while at a single exposure Istradefylline or AIH alone induce modest respiratory function, their effect is amplified when in combination.

In this proof-of-principle study, using a single administration, the PI will now determine how the combination works in patients and will evaluate relevant biological variables (e.g. baseline respiratory involvement, sleep- disordered breathing, disease severity, phenotype, etc) influence the levels of respiratory function. Although safety has not been done in vulnerable ALS patients, this study is OK as it will focus on single administrations of istradefylline before starting future studies of repetitive, daily dosing. Therefore, there are no concerns.

SCIENTIFIC MERIT

List the strengths and weaknesses of the proposed trial and how they may impact your decision to fund the proposal. Is the design of the study adequately developed and appropriate to achieve the aims of the study? Please comment on whether the proposed clinical outcomes measures and biomarker endpoints will provide a clear answer (positive or negative) on the trial results. Use this section to provide any specific feedback or raise concerns in the clinical trial narrative around trial period, trial population, inclusion/exclusion criteria, dose selection, sample size, biomarker strategy or schedule of activities. Are there novel tools, patient-centric endpoints or statistical approaches proposed that would increase success and improve efficiency of the trial?:

This is a straightforward Phase 1/II- proof-of-principle study.

For the most part, the study is based on a logical rationale. Although as indicated above, it would have been nice if the PIs addressed the controversial literature regarding the role of A2AR in ALS and the use of antagonists vs agonists in disease, the rationale is well explained and makes sense. Overall, the project proposes an interesting strategy to preserve respiratory function and independent breathing as long as possible in ALS patients. With the lack of effective treatments and paucity of exciting therapeutic targets at the horizon for sALS, any approach that delays disease progression and helps patients live independently is important.

The design of the study is appropriate. Outcome measures and endpoints are defined to accurately provide a clear answer for this proofreads-of-preinciple study.

The primary endpoint of safety (treatment differences in the rate of adverse events) is straightforward and seems appropriately powered.

The primary functional outcome looking at change in resting tidal volume 4- and 6- hours post-administration of istradefylline is also appropriate.

Secondary outcome measures, looking at acute changes in lab tests and vital signs and secondary functional measures (respiratory assessments etc) are also appropriate.
The PIs are aware of the potential side effects of the drug.
Sample size considerations seem adequate.

The group is well positioned to do this study and the PI is in the favorite position to have already performed AIH and AIH+caffeine in ALS patients. 8 patients underwent AIH.

TEAM/LEADERSHIP/STUDY SITES
Are the qualifications of the PI, key personnel, and collaborators/consultants appropriate to perform the proposed trial. Is the leadership and governance of the trial clearly described? Is there rationale provided for the selected trial sites? Do you recommend if a particular expertise is needed to the overall team structure (e.g. ALS trialist, statistician)?:
The PI and the group seem to have all the necessary expertise to conduct this pilot study.

BUDGET CONSIDERATIONS
Please comment on the overall budget and whether you think the budget is justified based on the proposed trial?:
Appropriate

FUTURE DRUG DEVELOPMENT
Does the commercialization and business plan provide a clear path forward for this particular drug program? If the proposed trial is funded and is successful, will it provide a clear go-no go decision to move forward into larger clinical trials? Are there partnership models or funding partners described that increase your confidence in the feasibility and viability of this program going forward?:
As stated above, if successful, this straightforward study has the potential to bring forward a useful strategy especially for early stage patients to delay mechanical ventilation and maintain independent breathing as long as possible.

PATIENT EXPERIENCE
Please comment on the strengths or weaknesses on patient engagement, recruitment and retention strategies described in the proposed trial.:
Well defined inclusion and exclusion criteria.

There is confidence that recruitment and retention will go as plan. Although intense, this is a short study, patients' engagement should not be a problem.

SUMMARY OF RECOMMENDATIONS
Please provide comments on how the proposal can be improved or strengthened. This feedback will be especially important to applicants who may not get funded through this opportunity and could help them secure funding from other agencies or in the next CTA cycle.:
No comments

Reviewer Role: Reviewer (775164)

SUMMARY OF GRANT PROPOSAL

Please provide a brief synopsis of the clinical trial and the overall goals of the proposal.:
Adenosine 2A receptor (A2AR) expression is dense in motor neurons. ALS models suggest that A2ARs accompanies motor neuron death.

The FDA approved A2AR antagonist, Istradefylline, improves motor neuron survival.

When combined with AIH therapy - a therapy used to stimulate 5-HT signaling to promote breathing - Istradefylline augments motor neuron function and breathing in ALS.

The objective of this proposal is to evaluate whether Istradefylline alone or in combination with AIH therapy improves breathing in ALS patients.

BIOLOGY

Please comment on the rationale/choice for the drug target/pathway and how relevant and important you think it is in ALS. Is there sufficient rationale provided for the biological links of the target to the disease?:
No issues raised with biological approach - this seems fundamentally sound. The preclinical and clinical data cited in the proposal supports the overall hypothesis of the proposal.

STRENGTH OF PRECLINICAL OR CLINICAL DATA

Please comment on the preclinical package provided for the therapeutic. Do the preclinical data on pharmacology, efficacy, PK/PD, safety/toxicology, etc. justify moving the therapeutic to the clinical stage? If the therapeutic has been tested in the clinic before, are there adequate safety, PK, target engagement data that supports further clinical development of this drug.:
No issues with biological hypothesis as described. Initial POC data suggests that this approach should significantly facilitate breathing and respiratory motor function. A strength of this work is that Istradefylline has confirmed safety (at the proposed doses) in elderly subjects. The preliminary/initial POC data suggests that the proposed approach of adding Istradefylline to AIH therapy should significantly reduce breathing issues in ALS patients. This is a safe and well tolerated drug in elderly subjects and PD patients and no untoward AEs are likely.

SCIENTIFIC MERIT

List the strengths and weaknesses of the proposed trial and how they may impact your decision to fund the proposal. Is the design of the study adequately developed and appropriate to achieve the aims of the study? Please comment on whether the proposed clinical outcomes measures and biomarker endpoints will provide a clear answer (positive or negative) on the trial results. Use this section to provide any specific feedback or raise concerns in the clinical trial narrative around trial period, trial population, inclusion/exclusion criteria, dose selection, sample size, biomarker strategy or schedule of activities. Are there novel tools, patient-centric endpoints or statistical approaches proposed that would increase success and improve efficiency of the trial?:
The sample size calculations were appropriately justified.

The 20 mg dose selection very well supported and justified – this is an active dose in PD patients and a dose that has been show to occupy A2ARs in the brain.

Can the authors provide any evidence that tolerance does not develop – either clinical or preclinically? I suspect this is not a major concern given the antagonist nature of this proposal but still may be worth understanding the effects of repeated dosing (which they propose to do next following this study)

TEAM/LEADERSHIP/STUDY SITES
Are the qualifications of the PI, key personnel, and collaborators/consultants appropriate to perform the proposed trial. Is the leadership and governance of the trial clearly described? Is there rationale provided for the selected trial sites? Do you recommend if a particular expertise is needed to the overall team structure (e.g. ALS trialist, statistician)?:
Very good team of ALS breathing researchers and A2AR pharmacology expert (Mitchell). Similarly, the team included the appropriate operations / drug dispensary / project management oversight required to successfully conduct and complete the proposed study. Overall, no issues with the team or facilities as described in the proposal. There is also a good outline for study governance and how to move this work forward at the Univ. of Florida.

This team also has experience in retaining ALS patients.

One suggestion to accelerate the proposed study timelines would be the addition of another clinical site.

BUDGET CONSIDERATIONS
Please comment on the overall budget and whether you think the budget is justified based on the proposed trial?:
No major issues with the proposed budget – appropriately justified budget both in terms of financial support for people and capital purchases.

FUTURE DRUG DEVELOPMENT
Does the commercialization and business plan provide a clear path forward for this particular drug program? If the proposed trial is funded and is successful, will it provide a clear go-no go decision to move forward into larger clinical trials? Are there partnership models or funding partners described that increase your confidence in the feasibility and viability of this program going forward?:
While this is a very well-written proposal, there is nothing new proposed that would warrant new intellectual property. Thus, if successful (and this reviewer believes that this proposal is likely to find a significant improvement in breathing), it is unclear who would marshal this combination forward – would it be the University of Florida or Kyowa Kiring Co or a yet-to-be-identified pharma partner? It would be a difficult approach to continue to advance this project through non-dilutive sources – perhaps the authors could provide more narrative describing their plans to move this project forward to commercialization should these results yield positive results.

Minor – this is no discussion that I could easily find related to publishing the results in various peer-reviewed journals.

PATIENT EXPERIENCE
Please comment on the strengths or weaknesses on patient engagement, recruitment and retention strategies described in the proposed trial.:
The inclusion of co-primary endpoints of safety and breathing function is a strength of the clinical trial plan. Could the age range be shortened (say 50-70 yrs) to get a more precise breathing issues in these patients – similarly, suggest stratifying (if possible) for gender.

Overall, the clinical measurements appear to be normal and within the range of what would be expected; however, it does seem like a long day for patients (up to 6 hours, likely more with travel, etc). This is only once per week so maybe a minor point, but I worry about patient fatigue.

SUMMARY OF RECOMMENDATIONS

Please provide comments on how the proposal can be improved or strengthened. This feedback will be especially important to applicants who may not get funded through this opportunity and could help them secure funding from other agencies or in the next CTA cycle.:

This proposal could be strengthened in the following ways:

1 - Only include ALS patients with existing breathing problems. This new inclusion criteria (with appropriate criteria) would be useful to confirm the overarching hypothesis (and presumably reduce baseline variability);

2 - Consider adding a second (or even a third) site to accelerate the pace of the study. This study seems to be very long (> 3 years) to get data in such a small (~ 18 patients) cohort of patients;

3 - Reduce the treatment groups to Istradefylline + sham and Istradefylline + AIH to confirm the hypothesis. This will take less time and less money and confirm your hypothesis.

Reviewer Role: Reviewer (777647)

SUMMARY OF GRANT PROPOSAL

Please provide a brief synopsis of the clinical trial and the overall goals of the proposal.:

Acute intermittent hypoxia (AIH) is a well-characterized stimulus to facilitate respiratory motor function in ALS animal models and in humans with chronic spinal injuries. AIH strengthens spinal synaptic pathways to spared motor neurons, producing respiratory motor facilitation (rMF) via serotonin-dependent signalling mechanisms explored extensively in the phrenic motor system of rats.

In the SOD1G93A transgenic rat model of ALS, even a single AIH session facilitates rMF via this serotonergic signaling pathway.

Neuromodulatory regulation by adenosine influences activity of glutamatergic synapses, and A2AR's specifically promote synaptic plasticity and neurotransmission. However, dysregulation of A2AR's appears in ALS . A2AR's are prevalent, particularly in motor neurons. It has been shown that excessive adenosine induces motor neuron damage, but motor neuron-astroglial survival and limb strength can be preserved with A2AR antagonism.

Istradefylline is a selective adenosine 2A receptor (A2AR) inhibitor. Istradefylline is marketed commercially by Kyowa Kirin Inc. as NOURIANZä and approved as adjunctive therapy in adults with Parkinson's disease. The safety of NOURIANZ TM is established with few, typically minor, side effects.

The A2AR -mediated signaling cascade can compete with, and undermine, AIH-induced, serotonin-dependent rMF.

In SOD1G93A rats, istradefylline removed adenosine mediated constraints and consequently enhanced AIH induced respiratory motor plasticity. In a rodent model of phrenic motor neuron death, A2AR antagonism also promoted motor neuron survival.

The applicant's preliminary data have shown in healthy adults that single adenosine antagonism or AIH exposures induce modest rMF, but the effect is amplified with a combinatorial approach.

The applicants recently completed the first trial of AIH in human ALS. When compared to an identical-appearing sham intervention, AIH significantly enhanced tidal volume, minute ventilation, and collective respiratory muscle activation of patients.

The proposal is to test the hypothesis that single doses of istradefylline are safe and acutely amplify respiratory function, and respiratory motor facilitation is further enhanced when paired with acute intermittent hypoxia.

They propose a repeated measures study design incorporating a double-blinded, placebo controlled, randomized, and counter-balanced trial, administered at a single site. Participation includes a screening and 4 individual study visits separated by 1 week. To evaluate separate and synchronous effects of singe exposures of istradefylline and AIH, each subject will under 4 test conditions: (1) istradefylline + AIH, (2) istradefylline + sham hypoxia, (3) placebo + AIH, and (4) placebo + sham hypoxia. Resting ventilation, respiratory muscle activation, maximal respiratory pressures and maximal grip force will be measured

BIOLOGY
Please comment on the rationale/choice for the drug target/pathway and how relevant and important you think it is in ALS. Is there sufficient rationale provided for the biological links of the target to the disease?:
The underlying biology justifying this proposed study is well-presented.

This study aims to investigate a therapeutic strategy to delay breathing problems in those with ALS utilizing istradefylline, a selective adenosine 2A receptor (A2AR) inhibitor, in combination with a therapeutic approach to promote respiratory plasticity known as acute intermittent hypoxia (AIH).

Data supporting each of istradefylline and AIH is provided and they appear to work through potentially complementary mechanisms.

Dysregulation of A2AR‘s appears in ALS and other neurodegenerative conditions and recent work illustrated that accumulation of extracellular adenosine induced motor neuron toxicity in C9orf72 and sporadic ALS. Selective A2AR inhibition attenuated the rate of toxic phrenic motor neuron death and preserved diaphragm activity. These effects have been observed in numerous models of ALS and do not appear to be genotype-specific.

AIH is a well-characterized stimulus to facilitate respiratory motor function in ALS animal models and humans with chronic spinal injuries (Rev in Gonzalez-Rothi 2015). AIH strengthens spinal synaptic pathways to spared motor neurons, producing respiratory motor facilitation (rMF) via serotonin-dependent signaling mechanisms. In the SOD1G93A transgenic rat model of ALS, even a single AIH session facilitates rMF via this serotonergic signaling pathway In contrast, elevated spinaladenosine triggers alternative cell signaling within phrenic motor neurons, via A2AR activation. This distinct A2AR -mediated signaling cascade competes with, and undermines, AIH-induced, serotonin-dependent rMF (Fields 2017). In SOD1G93A rats, istradefylline removed adenosine-mediated constraints to AIH and consequently enhanced respiratory motor plasticity – this provides a good basis for trialling the therapeutic approaches together.

STRENGTH OF PRECLINICAL OR CLINICAL DATA
Please comment on the preclinical package provided for the therapeutic. Do the preclinical data on pharmacology, efficacy, PK/PD, safety/toxicology, etc. justify moving the therapeutic to the clinical stage? If the therapeutic has been tested in the clinic before, are there adequate safety, PK, target engagement data that supports further clinical development of this drug.:
The applicants have preliminary data from healthy adults indicating that single adenosine antagonism or AIH exposures induce modest rMF, but the effect is amplified with a combinatorial approach

Both approaches have been well-used in clinical practice and are considered safe.

Istradefylline is marketed commercially by Kyowa Kirin Inc. as NOURIANZä and approved as adjunctive therapy to decrease “off time,” in adults with Parkinson’s disease (PD) taking levodopa/carbidopa. The safety of NOURIANZ TM is established in PD and unaffected elderly subjects, with few, typically minor, side effects.

Istradefylline has not been tested against ALS in humans.

AIH is simple to apply and well tolerated by most individuals. In over a dozen studies of humans with chronic spinal cord injury published to date, AIH protocols similar to that proposed here have not been associated with any adverse events. The applicants recently completed the first trial of AIH in human ALS. When compared to an identical-appearing sham intervention, AIH showed some moderate improvements in breathing. Importantly, the intervention was well-tolerated, free of treatment-related adverse effects, and the 23 participants typically could not distinguish the two interventions

SCIENTIFIC MERIT
List the strengths and weaknesses of the proposed trial and how they may impact your decision to fund the proposal. Is the design of the study adequately developed and appropriate to achieve the aims of the study? Please comment on whether the proposed clinical outcomes measures and biomarker endpoints will provide a clear answer (positive or negative) on the trial results. Use this section to provide any specific feedback or raise concerns in the clinical trial narrative around trial period, trial population, inclusion/exclusion criteria, dose selection, sample size, biomarker strategy or schedule of activities. Are there novel tools, patient-centric endpoints or statistical approaches proposed that would increase success and improve efficiency of the trial?:
The study design seems sound with group sizes sufficient to answer the questions beings asked. The outcomes are simple and with a short-term study – variation is less likely.
The treatment will need to be undertaken on a regular basis for the term of the disease (possibly years). Is there any information around tolerability of Istradefylline long-term? Also will resistance build up for either Istradefylline or AIH?
The study will also test the viability of RMCA as a biomarker of breathing function in ALS which they suggest may be more sensitive than EMG and that the RMCA can serve as an “early warning system” to detect impending ventilatory insufficiency. The applicants studies RMCA in their initial AIH trial in ALS and showed RMCA measured functional changes induced by AIH.
Additional tests will also be performed on consenting subjects to identify factors in variation in AIH response. This could also provide more information around how to best apply AIH. It is not clear however wither participant numbers will be sufficient for this extension to provide useful information.

TEAM/LEADERSHIP/STUDY SITES
Are the qualifications of the PI, key personnel, and collaborators/consultants appropriate to perform the proposed trial. Is the leadership and governance of the trial clearly described? Is there rationale provided for the selected trial sites? Do you recommend if a particular expertise is needed to the overall team structure (e.g. ALS trialist, statistician)?:
The team includes good experience across the range of technical knowledge required for the different aspects of this study including clinical, allied health and EMG.

BUDGET CONSIDERATIONS
Please comment on the overall budget and whether you think the budget is justified based on the proposed trial?:
Costs are well-described and justified.

FUTURE DRUG DEVELOPMENT

Does the commercialization and business plan provide a clear path forward for this particular drug program? If the proposed trial is funded and is successful, will it provide a clear go-no go decision to move forward into larger clinical trials? Are there partnership models or funding partners described that increase your confidence in the feasibility and viability of this program going forward?:

The company, Kyowa Kirin, making istradefylline are engaged in the project and have expressed interest in taking the project forward to more extensive chronic treatment trials if this initial trial proves positive. There is no funding currently in place for further trials so this will need to be sought through grants or industry funding.

PATIENT EXPERIENCE

Please comment on the strengths or weaknesses on patient engagement, recruitment and retention strategies described in the proposed trial.:

Patient engagement, recruitment and retention have all been addressed suitably in the application.
Different communication strategies have been considered as have providing Spanish, Portuguese and English language options.
Patient recruitment will be aided by a wide recruitment net.
Flexible appointment scheduling will be offered. The trial should be short-term as well to minimise participants being excluded from other trial opportunities.

SUMMARY OF RECOMMENDATIONS

Please provide comments on how the proposal can be improved or strengthened. This feedback will be especially important to applicants who may not get funded through this opportunity and could help them secure funding from other agencies or in the next CTA cycle.:

This is a well-designed and well-justified trial.
No major criticisms
